# Targeted isolation of antitubercular cycloheptapeptides and an unusual pyrroloindoline-containing new analog, asperpyrroindotide A, using LC–MS/MS-based molecular networking

**DOI:** 10.1007/s42995-022-00157-8

**Published:** 2023-01-20

**Authors:** Yi-Qian Han, Qun Zhang, Wei-Feng Xu, Yang Hai, Rong Chao, Cui-Fang Wang, Xue-Mei Hou, Mei-Yan Wei, Yu-Cheng Gu, Chang-Yun Wang, Chang-Lun Shao

**Affiliations:** 1 Key Laboratory of Marine Drugs, the Ministry of Education of China, School of Medicine and Pharmacy, Ocean University of China, Qingdao, 266003 China; 2 State Key Laboratory for Chemistry and Molecular Engineering of Medicinal Resources, School of Chemistry and Pharmaceutical Sciences, Guangxi Normal University, Guilin, 541004 China; 3 College of Food Science and Engineering, Ocean University of China, Qingdao, 266003 China; 4 Syngenta Jealott’s Hill International Research Centre, Bracknell, Berkshire RG42 6EY UK; 5 Laboratory for Marine Drugs and Bioproducts, Qingdao National Laboratory for Marine Science and Technology, Qingdao, 266237 China

**Keywords:** Marine natural product, MS/MS-based molecular networking, Cycloheptapeptides, Semi-synthesis, Anti-tubercular activity

## Abstract

Further insights on the secondary metabolites of a soft coral-derived fungus *Aspergillus versicolor* under the guidance of MS/MS-based molecular networking led to the isolation of seven known cycloheptapeptides, namely, asperversiamides A–C (**1**–**3**) and asperheptatides A–D (**4**–**7**) and an unusual pyrroloindoline-containing new cycloheptapeptide, asperpyrroindotide A (**8**). The structure of **8** was elucidated by comprehensive spectroscopic data analysis, and its absolute configuration was determined by advanced Marfey’s method. The semisynthetic transformation of **1** into **8** was successfully achieved and the reaction conditions were optimized. Additionally, a series of new derivatives (**10**−**19**) of asperversiamide A (**1**) was semi-synthesized and their anti-tubercular activities were evaluated against *Mycobacterium tuberculosis* H37Ra. The preliminary structure−activity relationships revealed that the serine hydroxy groups and the tryptophan residue are important to the activity.

**Supplementary Information:**

The online version contains supplementary material available at 10.1007/s42995-022-00157-8.

## Introduction

Tuberculosis (TB), a leading cause of mortality worldwide, is a life-threatening bacterial infection caused by *Mycobacterium tuberculosis* (Mtb) (Ardain et al. 2019). According to the WHO, the COVID-19 pandemic has reduced the access to TB diagnosis and treatment, and in 2020, the estimated number of deaths caused by TB increased to 1.5 million globally (Global tuberculosis report 2021). The first-line drugs used to treat TB include rifampicin, isoniazid, ethambutol and pyrazinamide. However, the widespread emergence of extensive drug-resistant tuberculosis (XDR-TB) and multidrug-resistant tuberculosis (MDR-TB) has increased the difficulty of TB treatment (Esmail et al. 2018). Meanwhile, the serious side effects of these drugs and long treatment period of TB have put enormous pressure on treatment worldwide (Slomski 2013). Therefore, the development of new anti-TB drugs has attracted significant attention.

Marine natural products (MNPs) have been recognized as a potential source of structurally novel and biologically active compounds that have yielded interesting chemical entities for drug discovery (Hai et al. 2021; Newman et al. 2020; Voser et al. 2022; Xu et al. 2022). More than 30 thousand new MNPs have been isolated from different marine organisms over the last 60 years (Blunt et al. 2018; Carroll et al. 2021). With the rapidly increasing numbers of natural products discovered each year, dereplication becomes critical to avoid re-isolating known compounds (Di et al. 2020). Molecular networking, a key strategy to visualize and annotate the chemical space in non-targeted mass spectrometry data, has become widely applied to the dereplication of marine natural products (Nothias et al. 2020).

**Fig. 1:**
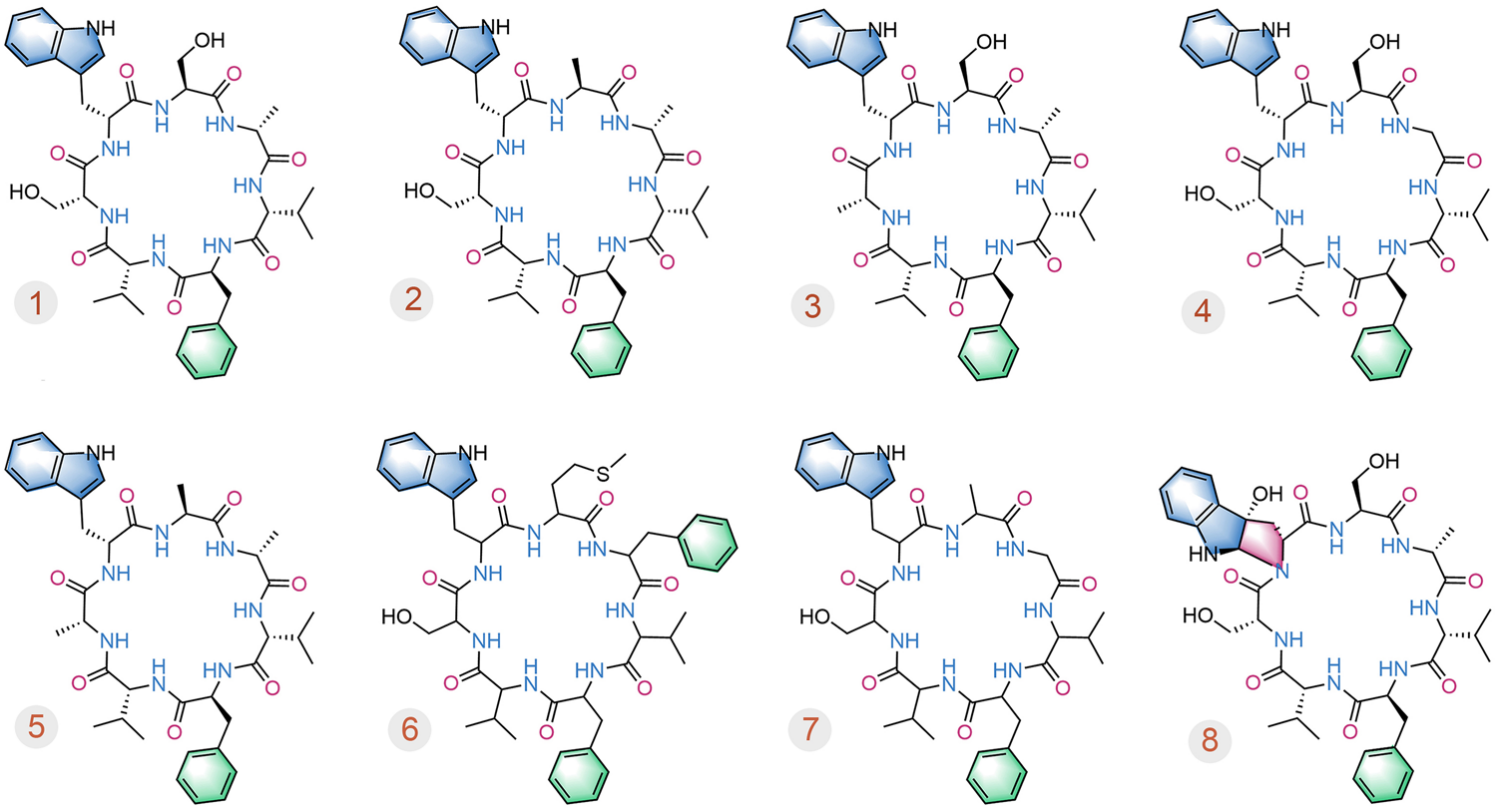
Structures of asperversiamides A–C (**1**–**3**), asperheptatides A–D (**4**–**7**) and asperpyrroindotide A (**8**)

In our previous research, a series of bioactive natural products were isolated from marine-derived fungi, and some active compounds have been synthesized (Guo et al. 2022; Jia et al. 2015; Shao et al. 2011, 2013; Xu et al. 2021) including the discovery of cyclohexadepsipeptides chrysogeamides A–G (Hou et al. 2019a), cycloheptapeptides asperversiamides A–C (**1**–**3**) (Hou et al. 2019b) and asperheptatides A–D (**4**–**7**) (Chao et al. 2021) in coral-derived fungi (Fig. 1) guided by LC–MS/MS-based molecular networking. Compounds **1**–**3** showed potent activity against *Mycobacterium marinum* with minimum inhibitory concentrations (MICs) of 23.4, 81.2, 87.5 µmol/L, respectively, which were equivalent to those of the positive controls, rifampin (19.0 µmol/L), streptomycin (20.1 µmol/L), and isoniazid (88.5 µmol/L). Compounds **4**–**7** showed anti-tubercular activity against *M. tuberculosis* H37Ra with the MICs at 100.0 µmol/L.

**Fig. 2:**
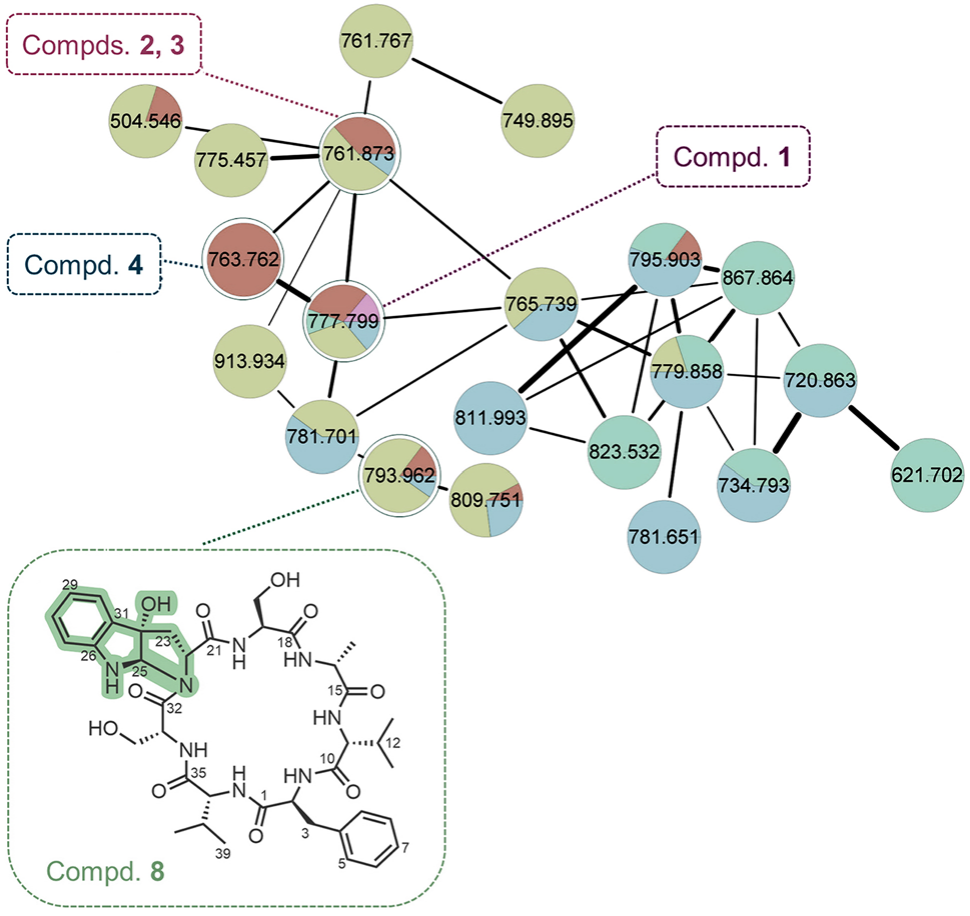
Structure of asperpyrroindotide A (**8**) containing the unusual motif pyrroloindoline, and the cluster corresponding to compounds of the asperversiamides family observed in the molecular networking

Further investigation on this type of cycloheptapeptides from the *A. versicolor* fungus guided by molecular networking led to the isolation of a new tricyclic pyrroloindoline-containing cycloheptapeptide, asperpyrroindotide A (**8**) (Fig. 2), together with the known analogs, asperversiamides A–C (**1**–**3**) and asperheptatides A–D (**4**–**7**). The complete structure of **8** including its absolute configuration was determined by comprehensive spectroscopic data and advanced Marfey’s method. The semi-synthesis of **8** from **1** was successfully achieved in one step and the reaction conditions were also investigated. Furthermore, a series of new derivatives (**10**−**19**) of asperversiamide A (**1**) were also semi-synthesized. Herein, we report the discovery and structure elucidation of the new cyclopeptide asperpyrroindotide A (**8**), and the preliminary structure–activity relationships of asperversiamide A (**1**) and its derivatives are discussed.

## Results and discussion

### Chemistry

The fungus *A. versicolor* (CHNSCLM-0063) was cultured on a rice solid medium. To optimize the fermentation conditions and discover new analogs, five amino acid precursors of asperversiamide A (**1**) were added to the rice solid medium. The production of the main compound asperversiamide A (**1**) was detected and the addition of alanine increased its yield (Supplementary Fig. S1). The rice solid medium was extracted with EtOAc. The fingerprints of the extracts showed no significant changes in the abundance of metabolites. However, the yields of some cycloheptapeptides (**1** and **2**) have been significantly improved. The extract was subjected to untargeted HPLC–MS/MS analysis, and a visualized molecular network was generated with the converted MS/MS data. The node with *m/z* 793.9 on the cycloheptapeptide network cluster was proposed to be a new cyclopeptide. The peak with *m/z* 793.9 was purified by silica gel, reversed-phase chromatography, and C18 RP HPLC and compound **8** was obtained.

**Fig. 3:**
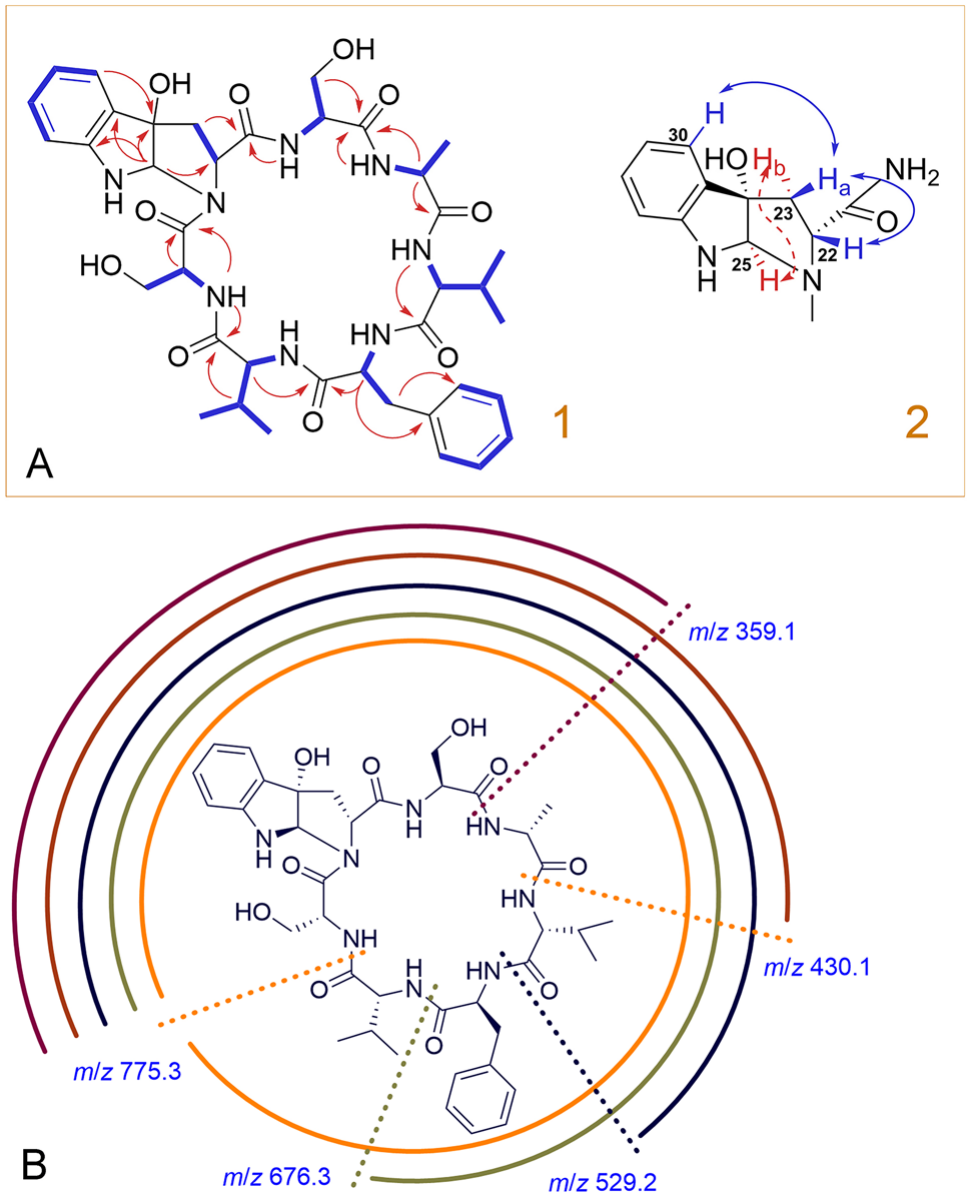
**A** **1** COSY and key HMBC correlations of **8**. **2** NOE correlations observed in the pyrroloindoline fragment of compound **8**. **B** ESI–MS/MS analysis of **8**

Asperpyrroindotide A (**8**) was obtained as a white solid. Its molecular formula was established as C_39_H_52_O_10_N_8_ with 18 degrees of unsaturation based on the HRESIMS, a [M + H]^+^ peak at *m/z* 793.3895 (calcd for C_39_H_53_O_10_N_8_^+^, 793.3879), and an [M + Na]^+^ peak at *m/z* 815.3707 (calcd for C_39_H_52_O_10_N_8_Na^+^, 815.3699). Its ^1^H NMR spectrum (Table 1) showed signals of six amide hydrogens, nine aromatic hydrogens, ten methine hydrogens, four methylene groups, and five methyl groups. The ^13^C NMR and HSQC data of **8** revealed 39 carbon signals, including seven carbonyl carbons, 12 aromatic carbons, one quaternary carbon, 10 methine carbons, four methylenes, and five methyls. Detailed analysis of its 1D and 2D NMR spectra revealed that **8** was a heptapeptides, containing one phenylalanine (Phe), one alanine (Ala), two valines (Val), two serines (Ser), and one unusual aromatic amino acid residue. The key HMBC correlations between Ala-NH/Ser_1_-CO, Val_2_-αH/Phe-CO, and Ser_2_-NH/Val_2_-CO (Fig. 3A, l), and the ESI–MS/MS fragment ions at *m/z* 676.3 (loss of Val_2_), *m/z* 529.2 (loss of Val_2_–Phe), *m/z* 410.2 (loss of Val_2_–Phe–Val_1_), and *m/z* 359.1 (loss of Val_2_–Phe–Val_1_–Ala) established the connectivity of the (NH) Ser–Ala–Val–Phe–Val–Ser (CO) fragment (Fig. 3B). The presence of a pyrroloindoline tricyclic residue was established by HMBC and TOCSY correlations in pyridine-*d*_5_ solution. TOCSY correlations between H-27 (*δ*_H_ 7.00), H-28 (*δ*_H_ 7.33), H-29 (*δ*_H_ 6.97), H-30 (*δ*_H_ 7.57) and H-22 (*δ*_H_ 4.88), H-23 (*δ*_H_ 3.06, 3.18), and the HMBC correlations from H-23 to C-21 (*δ*_C_ 173.6), from H-25 (*δ*_H_ 6.42) to C-22 (*δ*_C_ 62.8), C-24 (oxygenated carbon, *δ*_C_ 88.6), C-26 (*δ*_C_ 151.4), and C-31 (*δ*_C_ 132.5), from H-30 to C-24, were in agreement with the presence of the pyrroloindoline residue. These signals accounted for 17 of 18 degrees of unsaturation, indicating the final degree of unsaturation arising from the cyclic nature of **8**.

**Table 1:** NMR spectroscopic data of asperpyrroindotide A (**8**)^*a*^

Position	*δ*_C_	*δ*_H_ (*J* in Hz)	HMBC	TOCSY
1	173.2			
2	56.2	5.39 (1H, dd, *J* = 15.2, 7.4 Hz)	1, 3, 4	2-NH, 3
3a	38.2	3.06 (1H, overlapped)	1, 2, 4, 5	2, 2-NH
3b		3.75(1H, dd, *J* = 14.0, 7.4 Hz)		
4	138.9			
5/9	130.1	7.38 (2H, d, *J* = 7.2 Hz)	3, 5, 7	6, 7
6/8	129.3	7.33 (2H, overlapped)		5, 7
7	127.4	7.26 (1H, t, *J* = 7.2 Hz)	5	5, 6
2-NH		9.84 (1H, d, *J* = 7.4 Hz)	1, 2, 3	2, 3
10	173.3			
11	61.7	4.92 (1H, overlapped)		11-NH, 12, 13, 14
12	31.8	2.62 (1H, m)	10, 11, 13, 14	11-NH, 11, 13, 14
13	20.0	0.98 (3H, d, *J* = 6.6 Hz)	11, 12, 14	11-NH, 11, 12, 14
14	19.0	1.07 (3H, d, *J* = 6.6 Hz)	11, 12, 13	11-NH, 11, 12, 13
11-NH		9.38(1H, d, *J* = 8.9 Hz)	10	11, 12, 13, 14
15	173.6			
16	50.2	4.98 (1H, m)	15, 17, 18	16-NH, 17
17	17.7	1.66(3H, d, *J* = 6.6 Hz)	15, 16	16, 16-NH
16-NH		8.96 (1H, d, *J* = 7.8 Hz)	18	16, 17
18	170.8			
19	57.9	5.13 (1H, m)		19-NH, 20
20a	62.5	4.27 (1H, dd, *J* = 10.6, 2.8 Hz)	18, 19	19, 19-NH
20b		4.61 (1H, dd, *J* = 10.6, 4.6 Hz)		
19-NH		10.03 (1H, d, *J* = 7.8 Hz)	19, 20, 21	19, 20
21	173.6			
22	62.8	4.88 (1H, overlapped)		23
23a	40.7	3.06 (1H, overlapped)	21, 22, 24, 31	22
23b		3.18 (1H, t, *J* = 11.4 Hz)		
24	88.6			
25	83.6	6.42 (1H, s)	22, 24, 26, 31	
26	151.4			
27	112.0	7.00 (1H, d, *J* = 8.0 Hz)		28, 29, 30
28	131.0	7.33 (1H, overlapped)		27, 29, 30
29	120.4	6.97 (1H, overlapped)		27, 28, 30
30	125.1	7.57 (1H, overlapped)	24, 26, 28	27, 28, 29
31	132.5			
32	173.18			
33	56.2	5.71 (1H, m)	32, 34	33-NH, 34
34	63.8	4.57 (2H, overlapped)		33, 33-NH
33-NH		9.21 (1H, d, *J* = 6.2 Hz)	32, 35	33, 34
35	173.14			
36	61.9	4.32 (1H, m)	1, 37, 38	36-NH, 37, 38, 39
37	30.0	2.14 (1H, m)	35, 36, 39	36, 36-NH, 38, 39
38	19.5	0.80 (3H, d, *J* = 6.5 Hz)	37, 36, 39	36, 36-NH, 37, 39
39	20.1	0.87 (3H, d, *J* = 6.5 Hz)	36, 37, 38	36, 36-NH, 37, 38
36-NH		9.02 (1H, br s)		36, 37, 38, 39

^*a*^Measured in pyridine-*d*_5_, 500 MHz for ^1^H NMR and 125 MHz for ^13^C NMR

The relative configuration of H-22 (*δ*_H_ 4.05), 24-OH and H-25 (*δ*_H_ 5.54) in the pyrroloindoline fragment of **8** was assigned by analyses of selective 1D NOE difference experiments in DMSO-*d*_6_ solution (Fig. 3A, 2, Supplementary Fig. S3). The irradiation of H-25 resulted in an enhancement of the signal for H-22, suggesting that H-22 and H-25 should be placed on the same side of ring. However, the enhancement of H-23a (*δ*_H_ 2.54) and H-23b (*δ*_H_ 2.35) simultaneously was observed when irradiating H-25, prompting us to irradiate H-23a and H-23b with the same irradiation condition. The irradiation of H-23a resulted in the enhancement of the signal for H-23b (integral arbitrarily assigned a value of 1), H-22 (integral value of 0.28), H-25 (integral value of 0.02), and H-30 (*δ*_H_ 7.28, integral value of 0.17), and the irradiation of H-23b resulted in the enhancement of the signal for H-23a (integral arbitrarily assigned a value of 1), H-22 (integral value of 0.13), H-25 (integral value of 0.06), and H-30 (integral value of 0.05), indicating that H-22 and H-30 exhibited an NOE intensity stronger to H-23a than H-23b, and H-25 exhibited an NOE intensity stronger to H-23b than H-23a. H-22 and H-30 were therefore closer to H-23a than H-23b, and H-25 was closer to H-23b than H-23a. Thus, H-22 and H-23a should be *β*-oriented, and H-23b, 24-OH, and H-25 should be *α*-oriented. The pyrroloindole moiety can only be formed with a *cis* junction in either the *exo* or *endo* product when cyclization occurs (Lee et al. 2020; Liermann et al. 2009; Nakagawa et al. 1981; Ruiz-Sanchis et al. 2011); therefore, the *α*-orientation of 24-OH and H-25 was further confirmed.

To investigate the NOE intensity variation of H-23a and H-23b to H-22, H-22 was irradiated with different mixing times. Spectra were obtained in DMSO-*d*_6_ solution at 25 °C and 500 MHz with a relaxation delay of 1.0 s. The results of the intensity ratio for H-23a and H-23b are summarized in Table 2. It indicated that H-23a was closer to H-22 than H-23b under all the mixing times (variation from 50 to 1000 ms). The lower the mixing time (from 50 to 1000), the greater intensity difference, with the highest ratio of approximately 4:1 being attained.

**Table 2:** Ratio of H-23a/H-23b signal intensity in 1D NOE experiment by irradiation of H-22 from **1** under different mixing times

Entry	Mixing time (ms)	H-23a/H-23b ratio
1	50	1: 0.25
2	150	1: 0.41
3	250	1: 0.49
4	350	1: 0.46
5	600	1: 0.48
6	800	1: 0.54
7	1000	1: 0.59

The absolute configuration of the standard amino acid residues of **8** was determined by HPLC analysis of their acid hydrolysates derivatized with Marfey’s reagent (N_*α*_-(2,4-dinitro-5-fluorophenyl)-L-alalinamide, L-FDAA) (Marfey 1984). The derivatives were identified by comparison of their retention times in HPLC analyses with those of standards (Supplementary Fig. S4), confirming the presence of L-Phe (105.33 min), D-Val (102.23 min), D-Ala (64.35 min), D-Ser (33.77 min), and L-Ser (32.51 min) in **8**. The location of the L and D-Ser residues, and the absolute configuration of the pyrroloindoline residue could not be confirmed by standard Marfey’s method. Originally, the common biosynthetic pathway with asperversiamide A (**1**) was considered to speculate the location of L and D-Ser, and the *R* configuration at C-22 in **8**, according to the absolute configuration (Hou et al. 2019b). To further confirm the above result, we have performed the semisynthesis of asperpyrroindotide A (**8**) starting from asperversiamide A.

**Fig. 4:**
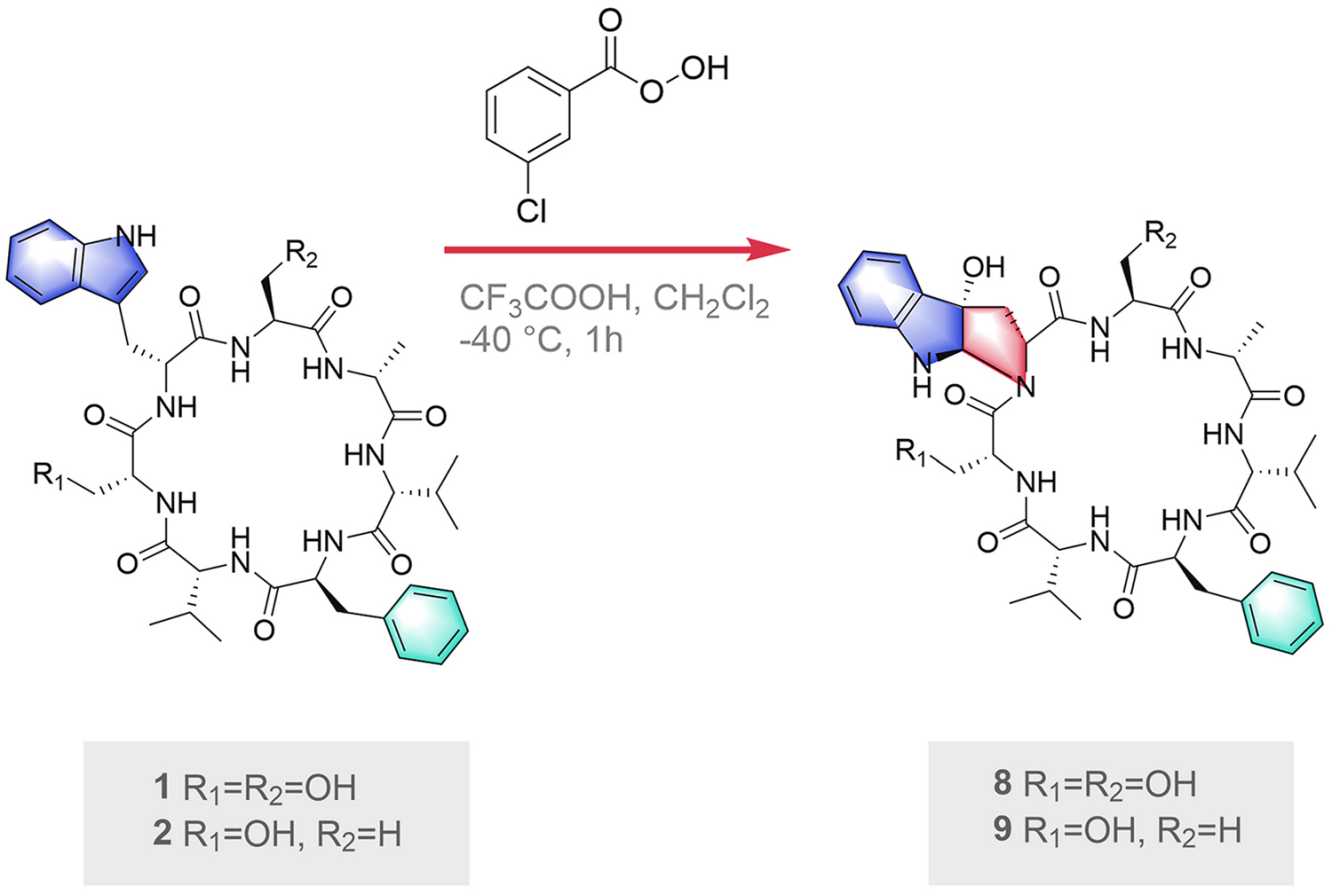
Semi-synthesis strategy for semi-synthesis of **8** and **9**

**Fig. 5:**
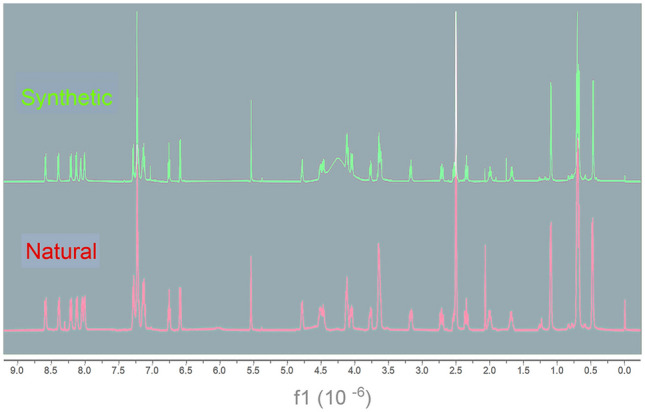
^1^H NMR spectra of natural and synthetic **8**

The semi-synthesis of the pyrroloindoline fragment was achieved by reported methods (Adhikari et al. 2016; Kitajima et al. 2006). Meta-chloroperoxybenzoic acid (*m*-CPBA) and trifluoroacetic acid (TFA) were employed to provide C3-hydroxypyrroloindoline in the cycloheptapeptide from the previously reported asperversiamide A (Fig. 4). The low yield of the target product (entry 1) prompted us to optimize the reaction (Table 3). Initially, we tried the reaction with different reaction solvents in the presence of 2 equiv of *m*-CPBA and 8 equiv of TFA at – 40 ℃ for 1 h. However, no desired product was detected (entry 2–4). Next, we increased the number of equivalents of *m*-CPBA. As a result, good yields of product were observed (entry 6 and 7) when more than 6 equiv of *m*-CPBA was added. With the optimized conditions (entry 6), 6.0 mg was synthesized. Its ^1^H NMR spectrum of the oxidation product was identical to that of the natural product, asperpyrroindotide A (**8**) (Fig. 5). Thus, the location of L and D-Ser and the absolute configuration at C-22 was confirmed. Finally, the absolute configuration of **8** was established as 2*S*, 11*R*, 16*R*, 19*S*, 22*R*, 24*S*, 25*R*, 33*R*, 36*R*. In addition, the semi-synthesis of the pyrroloindoline analog of asperversiamide B (**2**) was also carried out under these reaction conditions to obtain the derivative **9**.

**Table 3:** Optimization of reaction for the semi-synthesis of asperpyrroindotide A (**8**)^a^

Entry	Solvent	Equiv ^b^	Yield^c^/%
1	Dichloromethane	2	4.9
2	Ethanol	2	0
3	Acetone	2	0
4	1,4-Dioxane	2	0
5	Dichloromethane	4	15.6
6	Dichloromethane	6	19.6
7	Dichloromethane	8	19.6

^a^8 Equiv trifluoroacetic acid (TFA) catalyst was used in all cases, and the reaction time was 1 h

^b^The equiv of meta-chloroperoxybenzoic acid (*m*-CPBA)

^c^The yield of asperpyrroindotide A (**8**)

Although there are many natural peptides described in the literature, only a few cyclo-peptides having the pyrroloindoline motif in natural products have been reported to date (Supplementary Fig. S5). There are representative cases, such as the cyclic hexadepsipeptide antibiotic NW-G01 from the actinomycete *Streptomyces alboflavus* (Guo et al. 2009), the cyclic hexadepsipeptide anti-influenza melicopteline C from the leaves of *Melicope pteleifolia* (Lee et al. 2020), the apoptosis inducer and antimicrobial dimeric cyclohexapeptide chloptosin from the actinomycete *Streptomyces* strain (Umezawa et al. 2000), and the nematicidal cyclic dodecapeptides omphalotins E–I from the basidiomycete *Omphalotus olearius* (Liermann et al. 2009). The current cycloheptapeptide asperpyrroindotide A (**8**) is the first pyrroloindoline-containing cyclo-peptide isolated from marine fungi.

**Fig. 6:**
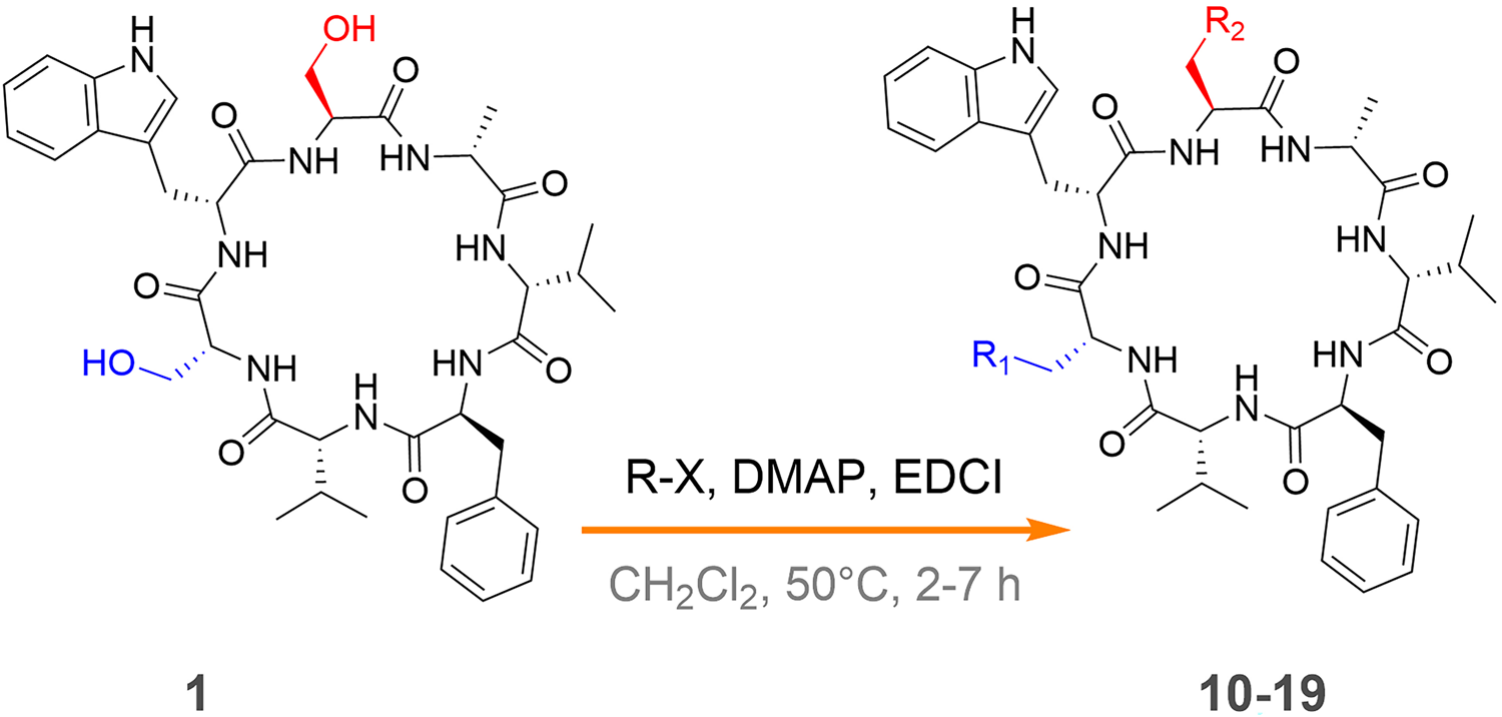
General strategy for semi-synthesis of asperversiamide A (**1**) derivatives

Our previous research results showed that some of the cycloheptapeptide analogs displayed anti-tubercular activity against *M. tuberculosis* H37Ra (Chao et al. 2021; Hou et al. 2019b). Compounds **8** and **9** were evaluated for their anti-tubercular activity against *M. tuberculosis* H37Ra. None of them showed obvious inhibitory activity at a concentration of 100 μg/mL. The result showed that the tryptophan residue in this class of cycloheptapeptides seems to be necessary for the anti-tubercular activity, and the formation of pyrroloindoline decreased the activity of asperversiamides A and B (**1** and **2**). Cinnamic acid as a vital element has shown potential for anti-TB drug discovery (Chao et al. 2021; Yang et al. 2020). To further investigate the structure–activity relationships (SAR), the related semisynthetic reagents were carefully considered and nitrogen-containing cinnamic acid analogs were selected. A group of new derivatives (**10**–**19**) of asperversiamide A (**1**) were semi-synthesized (Fig. 6). The structures of **10**−**19** were confirmed by extensive spectroscopic methods including ^1^H NMR, ^13^C NMR and HRESIMS (Supplementary Table S1). Compounds **1** and **8**−**19** were tested for their anti-tubercular activities against *M. tuberculosis* H37Ra (Supplementary Table S2). Their anti-tubercular activities were not significantly improved compared with our previously reported compounds **19**–**24**. Thus, from the results of **8**−**19** and all previously reported compounds, some notable structure−activity relationships (SARs) of this group of compounds can be drawn as a result of this research: compound **2**, containing the tryptophan residue, exhibited stronger anti-tubercular activity than **9** that has a pyrroloindoline moiety, obviously indicating that the tryptophan residue seems to be necessary for the anti-tubercular activity. Furthermore, the esterification of 20-OH and 34-OH did not appreciably improved the MIC values of most derivatives, implying that modifying the serine hydroxyl groups in these compounds had an unnoticeable effect on their antitubercular activity.

## Conclusion

In conclusion, we report herein the discovery and structure elucidation of an unusual pyrroloindoline-containing cycloheptapeptide, asperpyrroindotide A (**8**). The semisynthetic preparation of asperpyrroindotide A (**8**) from asperversiamide A (**1**) was successfully achieved in the optimized reaction conditions. In addition, a series of new derivatives (**10**−**19**) of asperversiamide A (**1**) were semi-synthesized and their anti-tubercular activities were evaluated. The preliminary structure−activity relationships indicated that the serine hydroxyl groups and the tryptophan residue in this type of cycloheptapeptides are important for antitubercular activity. Further studies on evaluating the anti-tubercular activity of cycloheptapeptides with different amino acid residues are in progress.

## Materials and methods

### General experimental procedures

UV spectra were recorded on a Beckman DU 640 spectrophotometer in MeOH solution. IR spectra were measured on a Nicolet Nexus 470 spectrophotometer in KBr disks. 1D and 2D NMR spectra were recorded on an Agilent 500 MHz DD2 spectrometer using TMS as an internal standard. ESIMS and HRESIMS spectra were performed on a Thermo Scientific LTQ Orbitrap XL spectrometer. HPLC–MS/MS were carried out on a Waters2695 HPLC instrument, coupled with an amaZon SL ion trap Mass spectrometer (Bruker), with a Xchange C18 column [(Acchrom Co.) 250 mm × 4.6 mm, 5 μm, 0.5 mL/min]. Semi-preparative HPLC was performed on a Hitachi L-2000 system (Hitachi Ltd.) using a C18 column [(Eka Ltd.) Kromasil 250 mm × 10 mm, 5 μm, 2.0 mL/min]. Silica gel (Qingdao Haiyang Chemical Group Co., 200–300 mesh), octadecylsilyl silica gel (YMC Co., Ltd., 45 − 60 μm).

### Fungal material, fermentation, extraction, and molecular networking

The fungal strain CHNSCLM-0063 was identified as *Aspergillus versicolor*. Its sequence data have been submitted to GenBank with the accession number MG736310. The procedures of fermentation, extraction, and molecular networking analysis were described in a previous report (Chao et al. 2021).

### Isolation

The EtOAc (EA) extract was subjected to silica gel vacuum liquid chromatography (VLC) and eluted by a gradient of petroleum ether (PE)/EA to EA and then EA/MeOH and afforded four sub-fractions (Fr.A − Fr.D). Fr.C (MeOH/EA 20%) was chromatographed by ODS column chromatography (MeOH/H_2_O, 40 − 100%) and yielded 20 sub-fractions (Fr.C1 − Fr.C20). Fr.C15 was purified by semi-preparative HPLC and eluted with MeCN/MeOH/H_2_O (25:25:50, v/v/v) to obtain compound **8** (3.5 mg).

Asperpyrroindotide A (**8**): white solid; UV (MeOH) *λ*_max_ (log *ε*) 200 (4.1), 230 (3.2), 293 (2.8) nm; IR (KBr) *ν*_max_ 3330, 1681, 1538 cm^–1^; ^1^H NMR (pyridine-*d*_5_, 500 MHz) and ^13^C NMR (pyridine-*d*_5_, 125 MHz), see Table 1; ^1^H NMR (DMSO-*d*_6_, 500 MHz): *δ* 8.58 (19-NH, d, *J* = 8.2 Hz, 1H), 8.39 (2-NH, d, *J* = 7.6 Hz, 1H), 8.21 (36-NH, d, *J* = 10.2 Hz, 1H), 8.12 (16-NH, d, *J* = 8.7 Hz, 1H), 8.04 (9-NH, d, *J* = 4.6 Hz, 1H), 8.01 (33-NH, d, *J* = 6.3 Hz, 1H), 7.32–7.19 (overlapped, 6H), 7.16–7.09 (overlapped, 2H), 6.76 (H-29, t, *J* = 7.4 Hz, 1H), 6.59 (H-27, d, *J* = 7.8 Hz, 1H), 5.54 (H-25, s, 1H), 4.79 (H-33, d, *J* = 5.6 Hz, 1H), 4.53 (H-2, m, 1H), 4.47 (H-16, m, 1H), 4.14–4.11 (overlapped, 2H), 4.05 (H-22, dd, *J* = 10.6, 6.2 Hz, 1H), 3.77 (H-34, dd, *J* = 10.6, 5.0 Hz, 1H), 3.64–3.61 (overlapped, 4H), 3.17 (H-3,dd, *J* = 13.7, 5.0 Hz, 1H), 2.72 (H-3, dd, *J* = 13.1, 10.6 Hz, 1H), 2.54 (H-23, m, 1H), 2.35 (H-23,t, *J* = 11.6 Hz, 1H), 2.00 (H-37, dd, *J* = 13.8, 7.1 Hz, 1H), 1.69 (H-12, dd, *J* = 13.7, 6.7 Hz, 1H), 1.10 (H_3_-17, d, *J* = 6.5 Hz, 3H), 0.73–0.65 (overlapped, 9H), 0.48 (H_3_-13, d, *J* = 6.6 Hz, 3H). ^13^C NMR (125 MHz, DMSO-*d*_6_) *δ* 171.4 (C-15), 170.9 (C-35), 170.8 (C-10), 170.7 (C-21), 170.6 (C-1), 169.7 (C-32), 168.7 (C-18), 149.7 (C-26), 137.8 (C-4), 130.7 (C-31), 129.7 (C-28), 129.1 (C-5, 9), 128.0 (C-6, 8), 126.2 (C-7), 124.0 (C-30), 118.7 (C-29), 109.9 (C-27), 86.6 (C-24), 81.1 (C-25), 61.8 (C-34), 60.9 (C-20), 60.6 (C-22), 59.9 (C-11), 59.3 (C-36), 55.9 (C-19), 54.8 (C-2), 54.0 (C-33), 47.6 (C-16), 40.0 (C-23), 36.7 (C-3), 30.7 (C-37), 28.6 (C-12), 19.2 (C-38), 18.9 (C-13), 18.2 (C-142), 17.7 (C-39), 17.5 (C-17). ESIMS/MS *m*/*z* 775.3 [M + H−H_2_O]^+^, *m*/*z* 676.3 [M + H−H_2_O−Val]^+^, *m*/*z* 529.2 [M + H−H_2_O−Val−Phe]^+^, *m*/*z* 430.17 [M + H−H_2_O−Val−Phe−Val]^+^, 359.1 [M + H−H_2_O−Val−Phe−Val−Ala]^+^; HRESIMS *m*/*z* 793.3895 [M + H]^+^ (calcd for C_39_H_53_O_10_N_8_^+^, 793.3879), *m*/*z* 815.3707 [M + Na]^+^ (calcd for C_39_H_52_O_10_N_8_Na^+^, 815.3699).

### Semisynthesis of asperpyrroindotide A (8)

The semisynthesis of asperpyrroindotide A (**8**) was conducted by reported methods (Adhikari et al. 2016). *m*-CPBA (39.9 mg, 0.23 mmol) was dissolved in 4 mL of dichloromethane. TFA (73.84 mg, 0.386 mmol) was added and the resulting mixture was stirred at rt for 1 h. The reaction was then cooled to − 40 °C and **1** (30.0 mg, 0.038 mmol) was added. After stirring at − 40 °C for 1 h, the reaction was quenched by the addition of saturated aqueous sodium sulfite solution (4 mL) and then was allowed to warm to room temperature. The mixture was extracted with CH_2_Cl_2_, and the organic extract was evaporated to dryness. The resulting residue was purified by RP HPLC, eluted with MeCN/MeOH/H_2_O (25:25:50, v/v/v), and pure compound **8** (6.0 mg) was obtained in the form of a white solid.

### Semisynthesis of compound 9

The semisynthesis of compound **9** was conducted using the same methods (Adhikari et al. 2016). *m*-CPBA (81.4 mg, 0.47 mmol) was dissolved in 8 ml of dichloromethane. TFA (150.77 mg, 0.786 mmol) was added and the resulting mixture was stirred at rt for 1 h. The reaction was then cooled to − 40 °C and **2** (60.0 mg, 0.079 mmol) was added. After stirring at − 40 °C for 1 h, the reaction was quenched by the addition of saturated aqueous sodium sulfite solution (8 mL) and then was allowed to warm to room temperature. The mixture was extracted with CH_2_Cl_2_, and the organic extract was evaporated to dryness. The resulting residue was purified by RP HPLC, eluted with MeCN/MeOH/H_2_O (28:28:44, v/v/v), and pure compound **9** (8.4 mg) was obtained in the form of a white solid.

### General procedure for the synthesis of 10−19

Compound **1** was dissolved in CH_2_Cl_2_ at 50 °C and stirred for 1 h. Then an appropriate amount of 4-dimethylaminopyridine (2 equiv, DMAP), 1-(3-dimethylaminopropyl)-3-ethyl-carbodiimid mono-hydrochloride (4 equiv, EDCI) and pyridine acid derivative analogs (4 equiv) were added and the solution was stirred at 50 °C until **1** was completely consumed by the analysis of HPLC. The mixture was extracted with water and the organic phase was separated. Then the CH_2_Cl_2_ layer was dried under reduced pressure, and the crude residue was purified by HPLC to afford compounds **10**−**19**.

### Antitubercular assay

Anti-mycobacterial activity was determined against *Mycobacterium tuberculosis* H37Ra (ATCC 25,177) in a microplate Alamar Blue assay system (Collins et al. 1997). The anti-tubercular drug rifampin was used as a positive control.

## Supplementary Information

Below is the link to the electronic supplementary material.

Supplementary file1 (DOCX 6663 KB)

## Data Availability

The data that supports the findings of this study are included in this published article (and its supplementary information file).
