# Supplementary material for: Targeted isolation of antitubercular cycloheptapeptides and an unusual pyrroloindoline-containing new analog, asperpyrroindotide A, using LC–MS/MS-based molecular networking
Source: Mar Life Sci Technol. 2023 Jan 20;5(1):85–93. doi: 10.1007/s42995-022-00157-8 (PMC9854410; doi:10.1007/s42995-022-00157-8)
Supplement: Supplementary file 1 — Supplementary file1 (DOCX 6663 KB) [file 42995_2022_157_MOESM1_ESM.docx]

**Targeted isolation of antitubercular cycloheptapeptides and an unusual pyrroloindoline-containing new analog, asperpyrroindotide A, using LC-MS/MS-based molecular networking**

Yi-Qian Han^1^ **·** Qun Zhang^1^ **·** Wei-Feng Xu*^,1,2^ **·** Yang Hai^1^ **·** Rong Chao^1^ **·** Cui-Fang Wang^1^ **·**Xue-Mei Hou^1^ **·** Mei-Yan Wei ^1,3^ **·** Yu-Cheng Gu^4^ **·** Chang-Yun Wang^1,5^ **·** Chang-Lun Shao*^,1,5^

1 Key Laboratory of Marine Drugs, the Ministry of Education of China, School of Medicine and Pharmacy, Ocean University of China, Qingdao 266003, China

2 State Key Laboratory for Chemistry and Molecular Engineering of Medicinal Resources, College of Chemistry and Pharmaceutical Sciences, Guangxi Normal University, Guilin 541004, China

3 College of Food Science and Engineering, Ocean University of China, Qingdao 266003, China

4 Syngenta Jealott’s Hill International Research Centre, Bracknell, Berkshire, RG42 6EY, UK

5 Laboratory for Marine Drugs and Bioproducts, Qingdao National Laboratory for Marine Science and Technology, Qingdao 266237, China

* Correspondence: shaochanglun@163.com (Chang-Lun Shao); xuweifeng_u@163.com (Wei-Feng Xu)

Yi-Qian Han and Qun Zhang contributed equally to this work.

**Compound 8**: White, amorphous powder; ^1^H NMR (500 MHz, Pyridine-*d*_5_): *δ* ppm 10.03 (1H, d, *J* = 7.9 Hz), 9.84 (1H, d, *J* = 6.9 Hz), 9.38 (1H, d, *J* = 8.9 Hz), 9.21 (1H, d, *J* = 6.2 Hz), 9.02 (1H, s), 8.95 (1H, d, *J* = 7.8 Hz), 7.58 – 7.55 (1H, overlapped), 7.40 – 7.36 (2H, overlapped), 7.33 (3H, t, *J* = 7.5 Hz), 7.28 – 7.24 (1H, overlapped), 7.01 – 6.94 (2H, overlapped), 6.43 (1H, s), 5.71 – 5.68 (1H, overlapped), 5.39 (1H, dd, *J* = 15.2, 7.4 Hz), 5.13 (1H, m), 4.98 (1H, m), 4.95 – 4.83 (2H, overlapped), 4.60 (1H, dd, *J* = 10.7, 4.6 Hz), 4.58 – 4.51 (2H, overlapped), 4.36 – 4.29 (1H, overlapped), 4.28 – 4.22(1H, overlapped), 3.75 (1H, dd, *J* = 14.0, 6.2 Hz), 3.18 (1H, t, *J* = 11.4 Hz), 3.10 – 3.01 (2H, overlapped), 2.61 (1H, m), 2.14 (1H, m), 1.66 (3H, d, *J* = 6.7 Hz), 1.06 (3H, d, *J* = 6.6 Hz), 0.98 (3H, d, *J* = 6.7 Hz), 0.86 (3H, d, *J* = 6.5 Hz), 0.80 (3H, d, *J* = 6.5 Hz); ^13^C NMR (125 MHz, Pyridine-*d*_5_): *δ* 173.6, 173.3, 173.2, 173.1, 173.1, 170.8, 151.4, 138.9, 132.5, 131.0, 130.1, 129.3, 127.4, 125.1, 120.4, 112.0, 88.6, 83.6, 63.8, 62.7, 62.5, 61.9, 61.7, 57.9, 56.2, 50.2, 40.7, 38.2, 31.8, 29.9, 20.1, 20.0, 19.5, 19.0, 17.7; HRESIMS *m*/*z* 793.3895 [M + H]^+^ (calcd for C_39_H_53_O_10_N_8_, 793.3879); 19.6% yield.

**Compound 9**: White, amorphous powder; ^1^H NMR (500 MHz, DMSO-*d*_6_): *δ* ppm 8.64 (1H, d, *J* = 7.8 Hz), 8.37 (1H, d, *J* = 7.8 Hz), 8.22 (1H, d, *J* = 9.9 Hz), 8.09 – 8.03 (2H, overlapped), 8.00 (1H, d, *J* = 6.2 Hz), 7.31 – 7.22 (6H, overlapped), 7.16 – 7.11 (2H, overlapped), 6.76 (1H, t, *J* = 7.4 Hz), 6.59 (1H, d, *J* = 7.8 Hz), 5.53 (1H, s), 4.78 (1H, dd, *J* = 12.0, 6.0 Hz), 4.54 – 4.50 (1H, overlapped), 4.50 – 4.45 (1H, overlapped), 4.14 – 4.08 (2H, overlapped), 3.90 (1H, dd, *J* = 10.7, 5.9 Hz), 3.76 (1H, dd, *J* = 10.7, 5.2 Hz), 3.65 – 3.63 (2H, overlapped), 3.18 (1H, m), 2.71 (1H, dd, *J* = 13.8, 10.3 Hz ), 2.54 – 2.52 (1H, overlapped), 2.34 (1H, t, *J* = 11.6 Hz), 2.00 (1H, m), 1.69 (1H, m), 1.20 (3H, d, *J* = 7.1 Hz), 1.08 (3H, d, *J* = 6.5 Hz), 0.71 – 0.67 (9H, overlapped), 0.48 (3H, d, *J* = 6.5 Hz); ^13^C NMR (150 MHz, DMSO-*d*_6_): *δ* 171.5, 171.0, 170.9, 170.9, 170.7, 170.1, 169.9, 149.8, 137.9, 130.7, 129.9, 129.2, 128.1, 126.3, 124.1, 118.8, 110.0, 86.7, 81.1, 61.8, 60.7, 60.0, 59.9, 54.8, 54.2, 48.6, 47.5, 36.8, 30.8, 30.7, 28.6, 19.2, 18.9, 18.3, 17.8, 17.6, 17.1; HRESIMS *m*/*z* 777.3921 [M + H]^+^ (calcd for C_39_H_53_O_9_N_8_, 777.3930); 14% yield.

**Compound 10**: White, amorphous powder; ^1^H NMR (400 MHz, DMSO-*d*_6_): *δ* ppm 10.83 (1H, s), 8.92 (1H, d, *J* = 8.1 Hz), 8.69 (2H, overlapped), 8.62 (1H, d, *J* = 4.6 Hz), 8.48 (1H, d, *J* = 7.6 Hz), 8.31 (1H, d, *J* = 8.4 Hz), 8.25 – 8.16 (2H, overlapped), 8.10 (1H, d, *J* = 7.8 Hz), 8.04 (1H, d, *J* = 5.4 Hz), 7.93 – 7.85 (2H, overlapped), 7.79 (1H, m), 7.61 (2H, overlapped), 7.49 (1H, d, *J* = 7.8 Hz), 7.27 (1H, d, *J* = 8.1 Hz), 7.25 – 7.19 (5H, overlapped), 7.15 (1H, m), 7.03 (1H, t, *J* = 7.5 Hz), 6.95 (1H, t, *J* = 6.9 Hz), 4.76 (1H, dd, *J* = 10.8, 3.9 Hz), 4.71 – 4.57 (3H, overlapped), 4.57 – 4.43 (4H, overlapped), 4.35 (1H, dd, J = 11.1, 3.9 Hz), 4.04 (1H, m), 3.78 (1H, dd, *J* = 7.48, 5.39 Hz), 3.18 (1H, dd, *J* = 13.7, 4.8 Hz), 3.09 (1H, dd, J = 14.9, 7.2 Hz), 2.99 (1H, dd, J = 14.9, 7.1 Hz), 2.73 (1H, dd, J = 13.8, 10.3 Hz), 1.92 (1H, m), 1.73 (1H, m), 0.95 (3H, d, *J* = 6.8 Hz), 0.69 (3H, d, *J* = 6.7 Hz), 0.63 (3H, d, *J* = 6.7 Hz), 0.59 (3H, d, *J* = 6.7 Hz), 0.48 (3H, d, *J* = 6.7 Hz); ^13^C NMR (150 MHz, DMSO-*d*_6_): *δ* 172.5, 171.7, 171.5, 171.3, 169.4, 168.6, 164.6, 164.5, 150.4, 150.3, 147.7 , 147.6 , 138.3, 137.8, 137.6, 136.6, 129.7, 128.6, 128.0, 127.7, 126.8, 126.2, 125.8, 124.0, 121.5, 118.8, 111.8, 109.8, 64.9, 64.0, 60.2 , 60.0, 55.5, 55.3, 52.6, 52.4, 48.7, 37.2, 30.9, 29.7 , 26.9, 19.6, 19.3, 18.8, 18.2, 18.1; HRESIMS *m*/*z* 987.4300 [M + H]^+^ (calcd for C_51_H_59_O_11_N_10_, 987.4359); 41% yield.

**Compound 11**: White, amorphous powder; ^1^H NMR (400 MHz, DMSO-*d*_6_): *δ* ppm 10.86 (1H, d, *J* = 2.4 Hz), 9.09 (1H, d, *J* = 1.3 Hz), 8.80 (1H, dd, *J* = 4.9, 1.7 Hz), 8.55 (1H, d, *J* = 4.4 Hz), 8.47 (2H, t, *J* = 7.0 Hz), 8.31 (1H, m), 8.25 (2H, t, *J* = 7.7 Hz), 8.18 (1H, d, *J* = 8.4 Hz), 8.04 (1H, d, *J* = 5.2 Hz), 7.55 (1H, d, *J* = 7.9 Hz), 7.47 (1H, dd, *J* = 8.1, 5.3 Hz), 7.32 (1H, d, *J* = 8.1 Hz), 7.26 – 7.17 (5H, overlapped), 7.15 (1H, m), 7.05 (1H, m), 6.96 (1H, t, *J* = 7.0 Hz), 4.79 (1H, dd, *J* = 10.6, 3.6 Hz), 4.73 – 4.57 (2H, overlapped), 4.56 – 4.40 (4H, overlapped), 4.18 (1H, m), 4.02 (1H, t, *J* = 8.8 Hz), 3.74 (1H, dd, *J* = 7.5, 5.3 Hz), 3.65 (1H, m), 3.42 (1H, m), 3.17 (1H, dd, *J* = 13.8, 4.7 Hz), 3.10 (1H, dd, *J* = 14.8, 7.2 Hz), 2.97 (1H, dd, *J* = 14.7, 7.4 Hz), 2.72 (1H, dd, *J* = 13.8, 10.5 Hz), 1.91 (1H, m), 1.75 (1H, m), 0.92 (3H, d, *J* = 6.8 Hz), 0.70 (3H, d, *J* = 6.7 Hz), 0.64 (3H, d, *J* = 6.7 Hz), 0.60 (3H, d, *J* = 6.7 Hz), 0.47 (3H, d, *J* = 6.7 Hz); ^13^C NMR (150 MHz, DMSO-*d*_6_): *δ* 172.4, 171.91, 171.6, 171.3, 171.3, 167.0, 169.1, 165.2, 154.3, 150.9, 138.4, 137.9, 136.61, 129.7, 128.6, 127.8, 126.8, 125.7, 124.2, 121.4, 118.9, 118.7, 111.8, 109.9, 63.8, 61.5, 60.4, 60.1, 56.2, 55.7, 55.3, 52.3, 48.7, 37.0, 30.8, 29.6, 26.9, 19.7, 19.2, 18.7, 18.3, 18.0; HRESIMS *m*/*z* 882.4123 [M + H]^+^ (calcd for C_45_H_56_O_10_N_9_, 882.4123); 21% yield.

**Compound 12**: White, amorphous powder; ^1^H NMR (400 MHz, DMSO-*d*_6_): *δ* ppm 10.84 (1H, d, *J* = 2.4 Hz), 9.08 (1H, dd, *J* = 8.2, 1.8 Hz), 8.91 (1H, d, *J* = 8.4 Hz), 8.81 (1H, dd, *J* = 4.9, 1.7 Hz), 8.77 (2H, dd, *J* = 4.8, 1.7 Hz), 8.55 (1H, d, *J* = 7.7 Hz), 8.37 (1H, d, *J* = 8.5 Hz), 8.32 (1H, m), 8.26 (2H, dd, *J* = 11.7, 7.8 Hz), 8.15 – 8.07 (2H, overlapped), 7.53 – 7.46 (2H, overlapped), 7.40 (1H, dd, *J* = 7.9, 5.0 Hz), 7.28 – 7.19 (6H, overlapped), 7.14 (1H, m), 7.02 (1H, m), 6.94 (1H, t, *J* = 7.0 Hz), 4.83 (1H, dd, *J* = 10.8, 3.6 Hz), 4.75 – 4.62 (3H, overlapped), 4.60 – 4.43 (4H, overlapped), 4.16 (1H, dd, *J* = 11.0, 3.5 Hz), 4.06 (1H, t, *J* = 8.8 Hz), 3.72 (1H, dd, *J* = 7.5, 5.0 Hz), 3.22 (1H, dd, *J* = 13.9, 4.5 Hz), 3.13 – 2.95 (2H, overlapped), 2.73 (1H, dd, *J* = 13.8, 10.8 Hz), 1.94 (1H, m), 1.68 (1H, m), 0.91 (3H, d, *J* = 6.7 Hz), 0.69 – 0.61 (6H, overlapped), 0.59 (3H, d, *J* = 6.7 Hz), 0.43 (3H, d, *J* = 6.7 Hz); ^13^C NMR (100 MHz, DMSO-*d*_6_): *δ* 172.5, 171.6, 171.5, 171.3, 171.1, 169.3, 168.5, 165.2, 164.8, 154.3, 154.1, 150.9, 150.9, 138.4, 137.7, 137.4, 136.5, 129.6, 128.5, 127.6, 125.7, 125.6, 124.1, 123.9, 121.4, 118.7, 111.8, 109.7, 65.0, 63.6, 60.7, 60.0, 55.7, 55.3, 52.3, 52.2, 48.6, 36.9, 31.1, 29.5, 26.8, 19.6, 19.1, 18.6, 18.2, 17.9; HRESIMS *m*/*z* 987.4336 [M + H]^+^ (calcd for C_51_H_59_O_11_N_10_, 987.4359); 33% yield.

**Compound 13**: White, amorphous powder; ^1^H NMR (400 MHz, DMSO-*d*_6_): *δ* ppm 10.87 (1H, d, *J* = 2.5 Hz), 8.97 (1H, d, *J* = 8.5 Hz), 8.82 (1H, d, *J* = 4.0 Hz), 8.76 – 8.72 (2H, overlapped), 8.64 – 8.51 (3H, overlapped), 8.38 (1H, d, *J* = 8.5 Hz), 8.25 (1H, d, *J* = 6.7 Hz), 8.12 (1H, d, *J* = 4.9 Hz), 7.91 – 7.83 (2H, overlapped) , 7.69 – 7.62 (2H, overlapped), 7.49 (1H, d, *J* = 7.8 Hz), 7.31 – 7.12 (8H, overlapped), 7.03 (1H, t, *J* = 7.5 Hz), 6.96 (1H, t, *J* = 7.3 Hz), 4.83 (1H, dd, *J* = 11.0, 4.0 Hz), 4.76 – 4.64 (3H, overlapped), 4.59 – 4.39 (4H, overlapped), 4.31 (1H, dd, *J* = 11.1, 3.4 Hz), 4.06 (1H, t, *J* = 8.8 Hz), 3.75 (1H, dd, *J* = 7.5, 5.0 Hz), 3.22 (1H, dd, *J* = 13.8, 4.6 Hz), 3.13 (1H, dd, *J* = 14.9, 8.0 Hz), 3.02 (1H, dd, *J* = 14.9, 6.8 Hz), 2.74 (1H, dd, *J* = 13.8, 10.7 Hz), 1.91 (1H, m), 1.71 (1H, m), 0.87 (3H, d, *J* = 6.7 Hz), 0.74 – 0.63 (6H, overlapped), 0.59 (3H, d, *J* = 6.7 Hz), 0.46 (3H, d, *J* = 6.7 Hz); ^13^C NMR (150 MHz, DMSO-*d*_6_): *δ* 172.6, 171.6, 171.3, 171.2, 169.3, 168.5, 165.1, 164.8, 151.0, 138.4, 137.1, 137.0, 136.6, 129.7, 128.6, 127.7, 126.8, 123.9, 123.4, 123.2, 121.6, 118.8, 118.7, 111.8, 109.7, 65.4, 64.2, 60.5, 60.2, 55.7, 55.4, 52.4, 52.2, 48.7, 36.9, 31.1, 29.6, 26.8, 19.6, 19.2, 18.7, 18.3, 18.1; HRESIMS *m*/*z* 987.4321 [M + H]^+^ (calcd for C_51_H_59_O_11_N_10_, 987.4359); 51% yield.

**Compound 14**: White, amorphous powder; ^1^H NMR (400 MHz, DMSO-*d*_6_): *δ* ppm 10.87 (1H, d, *J* = 2.4 Hz), 8.64 (1H, d, *J* = 3.5 Hz), 8.51 – 8.41 (3H, overlapped), 8.30 – 8.22 (2H, overlapped), 8.09 (1H, d, *J* = 8.8 Hz), 7.96 (1H, d, *J* = 5.6 Hz), 7.85 (1H, m), 7.76 (1H, d, *J* = 15.7 Hz), 7.67 (1H, d, *J* = 7.7 Hz), 7.56 (1H, d, *J* = 7.7 Hz), 7.41 (1H, dd, *J* = 7.7, 5.8 Hz), 7.32 (1H, d, *J* = 8.1 Hz), 7.27 – 7.18 (6H, overlapped), 7.15 (1H, m), 7.05 (1H, t, *J* = 7.5 Hz), 6.96 (1H, t, *J* = 7.3 Hz), 6.79 (1H, d, *J* = 15.7 Hz), 4.72 (1H, t, *J* = 5.6 Hz), 4.64 – 4.57 (2H, overlapped), 4.55 – 4.49 (2H, overlapped), 4.44 (1H, m), 4.14 (1H, m), 4.02 (1H, t, *J* = 8.8 Hz), 3.79 (1H, t, *J* = 6.5 Hz), 3.65 (1H, m), 3.43 (1H, m), 3.20 – 3.06 (2H, overlapped), 2.98 (1H, dd, *J* = 14.8, 7.3 Hz), 2.74 (1H, dd, *J* = 13.7, 10.1 Hz), 1.98 (1H, m), 1.79 (1H, m), 1.03 (3H, d, *J* = 6.8 Hz), 0.71 – 0.66 (6H, overlapped), 0.65 (3H, d, *J* =6.8 Hz), 0.50 (3H, d, *J* = 6.8 Hz); ^13^C NMR (150 MHz, DMSO-*d*_6_): *δ* 172.5, 171.8, 171.6, 171.30, 169.8, 169.4, 166.2, 152.6, 150.7, 144.8, 138.3, 137.8, 136.6, 129.7, 128.6, 127.8, 126.9, 125.7, 125.4, 124.2, 121.4, 121.2, 118.9, 118.7, 111.8, 109.9, 63.2, 61.5, 60.2, 59.9, 56.6, 55.5, 55.3, 52.4, 48.6, 37.1, 30.7, 29.6, 27.0, 19.6, 19.3, 18.7, 18.3, 18.1; HRESIMS *m*/*z* 908.4321 [M + H]^+^ (calcd for C_47_H_58_O_10_N_9_, 908.4301); 19% yield.

**Compound 15**: White, amorphous powder; ^1^H NMR (400 MHz, DMSO-*d*_6_): *δ* ppm 10.88 (1H, d, *J* = 1.9 Hz), 8.85 (1H, d, *J* = 8.0 Hz), 8.64 (2H, d, *J* =3.7 Hz), 8.61 (1H, d, *J* = 4.2 Hz), 8.52 (1H, d, *J* = 7.6 Hz), 8.32 – 8.24 (3H, overlapped), 8.07 (1H, m), 7.90 – 7.81 (2H, overlapped), 7.76 (1H, d, *J* = 15.7 Hz), 7.73 – 7.60 (3H, overlapped), 7.51 (1H, d, *J* = 7.9 Hz), 7.47 – 7.37 (2H, overlapped), 7.29 (1H, d, *J* = 8.1 Hz), 7.26 – 7.19 (5H, overlapped), 7.16 (1H, m), 7.03 (1H, t, *J* = 7.0 Hz), 6.95 (1H, t, *J* = 7.4 Hz), 6.81 (2H, dd, *J* = 15.7, 9.1 Hz), 4.69 – 4.57 (3H, overlapped), 4.57 – 4.44 (3H, overlapped), 4.37 (1H, dd, *J* = 11.1, 5.8 Hz), 4.14 (1H, dd, *J* = 11.1, 3.6 Hz), 4.06 (1H, t, *J* = 8.9 Hz), 3.76 (1H, dd, *J* = 7.4, 5.2 Hz), 3.24 – 2.97 (3H, overlapped), 2.74 (1H, dd, *J* = 13.8, 10.4 Hz), 1.98 (1H, m), 1.72 (1H, m), 1.23 (1H, s), 1.03 (3H, d, *J* = 6.7 Hz), 0.71 – 0.61 (9H, overlapped), 0.45 (3H, d, *J* = 6.7 Hz); ^13^C NMR (100 MHz, DMSO-*d*_6_): *δ* 172.6, 171.6, 171.5, 171.3, 171.1, 169.4, 168.4, 166.1, 166.0, 152.5, 150.6, 150.5, 144.7, 144.3, 138.3, 137.8, 136.5, 129.6, 128.5, 127.7, 126.6, 125.5, 125.4, 124.0, 121.6, 121.4, 121.1, 118.7, 111.8, 109.7, 64.2, 63.1, 60.3, 60.1, 55.5, 55.3, 52.8, 52.3, 48.5, 37.2, 31.0, 30.0, 26.7, 19.5, 19.2, 18.7, 18.3, 18.0; HRESIMS *m*/*z* 1039.4676 [M + H]^+^ (calcd for C_55_H_63_O_11_N_10_, 1039.4672); 59% yield.

**Compound 16**: White, amorphous powder; ^1^H NMR (400 MHz, DMSO-*d*_6_): *δ* ppm 10.89 (1H, d, *J* = 2.4 Hz), 8.90 – 8.82 (3H, overlapped), 8.65 – 8.56 (3H, overlapped), 8.50 (1H, d, *J* = 7.4 Hz), 8.27 (1H, d, *J* = 9.5 Hz), 8.21 (1H, d, *J* = 8.7 Hz), 8.16 (1H, d, *J* = 6.6 Hz), 8.14 – 8.05 (2H, overlapped), 7.95 (1H, d, *J* = 5.6 Hz), 7.78 (1H, d, *J* = 16.2 Hz), 7.66 (1H, d, *J* = 16.2 Hz), 7.50 (1H, d, *J* = 7.8 Hz), 7.48– 7.41 (2H, overlapped), 7.32 – 7.19 (6H, overlapped), 7.15 (1H, m), 7.01 (1H, t, *J* = 7.0 Hz), 6.93 (1zH, t, *J* = 7.4 Hz), 6.62 (2H, dd, *J* = 17.9, 16.2 Hz), 4.65 (1H, dd, *J* = 3.1 Hz, 9.0 Hz), 4.61 – 4.43 (6H, overlapped), 4.31 (1H, dd, *J* = 11.2, 6.5 Hz), 4.21 (1H, dd, *J* = 11.2, 3.7 Hz), 4.05 (1H, dd, *J* = 9.5, 7.5 Hz), 3.83 (1H, t, *J* = 6.3 Hz), 3.19 – 3.08 (2H, overlapped), 3.02 (1H, dd, *J* = 15.7, 7.8 Hz), 2.76 (1H, dd, *J* = 13.8, 10.1 Hz), 2.00 (1H, m), 1.78 (1H, m), 1.04 (3H, d, *J* = 6.8 Hz), 0.73 – 0.60 (9H, overlapped), 0.51 (3H, d, *J* = 6.8 Hz); ^13^C NMR (150 MHz, DMSO-*d*_6_) *δ* 172.7, 171.7, 171.5, 171.3, 169.6, 168.4, 165.9, 151.6, 150.3 ( × 2), 142.4, 142.2, 138.2, 136.5, 135.3, 135.1, 130.4, 130.3, 129.7, 128.6, 127.7, 126.8, 124.6, 124.5, 124.0, 121.5, 120.1, 119.9, 118.8, 111.8, 109.8, 64.1, 60.0, 59.7, 55.5, 55.3, 53.0, 52.3, 48.6, 37.1, 30.6, 29.9, 27.0, 19.4, 19.3, 18.8, 18.2, 18.1; HRESIMS *m*/*z* 1039.4652 [M + H]^+^ (calcd for C_55_H_63_O_11_N_10_, 1039.4672); 55% yield.

**Compound 17**: White, amorphous powder; ^1^H NMR (400 MHz, DMSO-*d*_6_): *δ* ppm 10.88 (1H, s), 9.03 (1H, dd, *J* = 4.2, 1.8 Hz), 8.83 (1H, d, *J* = 1.9 Hz), 8.63 (2H, dd, *J* = 12.0, 5.9 Hz), 8.52 (2H, dd, *J* = 11.0, 7.8 Hz), 8.29 (2H, d, *J* = 7.9 Hz), 8.25 – 8.17 (2H, overlapped), 8.06 – 7.99 (2H, overlapped), 7.63 (1H, dd, *J* = 8.3, 4.2 Hz), 7.55 (1H, d, *J* = 7.8 Hz), 7.32 (1H, d, *J* = 8.1 Hz), 7.29 – 7.18 (5H, overlapped), 7.15 (1H, m), 7.06 (1H, t, *J* = 7.3 Hz), 6.96 (1H, t, *J* = 7.4 Hz), 4.89 (1H, dd, *J* = 11.1, 4.4 Hz), 4.77 – 4.69 (2H, overlapped), 4.65 (1H, dd, *J* = 11.0, 4.8 Hz), 4.58 (1H, m), 4.48 (1H, m), 4.41 (1H, dd, *J* = 8.5, 6.6 Hz), 4.25 (1H, m), 4.03 (1H, t, *J* = 8.9 Hz), 3.77 – 3.66 (2H, overlapped), 3.47 (1H, m), 3.22 – 3.09 (2H, overlapped), 3.03 (1H, dd, *J* = 14.9, 7.4 Hz), 2.73 (1H, dd, *J* = 13.8, 10.5 Hz), 1.91 (1H, m), 1.76 (1H, m), 0.81 (3H, d, *J* = 6.7 Hz), 0.70 (3H, d, *J* = 6.7 Hz), 0.64 (3H, d, *J* = 6.6 Hz), 0.59 (3H, d, *J* = 6.6 Hz), 0.46 (3H, d, *J* = 6.7 Hz); ^13^C NMR (150 MHz, DMSO-*d*_6_) *δ* 172.8, 171.7, 171.6, 171.3, 171.2, 169.9, 169.4, 165.9, 153.5, 150.0, 138.4, 138.0, 136.6, 132.5, 129.7, 129.3, 128.6, 127.8, 127.7, 127.5, 126.8, 124.2, 122.9, 121.4, 118.8, 118.7, 111.8, 109.8, 63.5, 61.6, 60.5, 60.1, 56.6, 55.8, 55.4, 52.3, 48.6, 36.9, 30.8, 29.5, 26.9, 19.7, 19.2, 18.6, 18.3, 17.9; HRESIMS *m*/*z* 932.4276 [M + H]^+^ (calcd for C_49_H_58_O_10_N_9_, 932.4301); 23% yield.

**Compound 18**: White, amorphous powder; ^1^H NMR (400 MHz, DMSO-*d*_6_): *δ* ppm 10.88 (1H, d, *J* = 1.4 Hz), 9.09 (1H, d, *J* = 8.0 Hz), 9.06 – 9.00 (2H, overlapped), 8.87 –8.81 (2H, overlapped), 8.66 (1H, d, *J* = 1.9 Hz), 8.63 – 8.52 (3H, overlapped), 8.39 (1H, d, *J* = 8.9 Hz), 8.29 (1H, d, *J* = 9.7 Hz), 8.23 (1H, dd, *J* = 8.9, 2.0 Hz), 8.15 (1H, dd, *J* = 8.8, 1.9 Hz), 8.11 (1H, d, *J* = 5.8 Hz), 8.06 (1H, d, *J* = 8.8 Hz), 8.02 (1H, d, *J* = 8.8 Hz), 7.96 (1H, d, *J* = 5.5 Hz), 7.68 – 7.58 (2H, overlapped), 7.48 (1H, d, *J* = 7.9 Hz), 7.31 – 7.20 (7H, overlapped), 7.16 (1H, m), 7.01 (1H, t, *J* = 7.5 Hz), 6.93 (1H, t, *J* = 7.4 Hz), 4.86 – 4.77 (2H, overlapped), 4.75 (1H, m), 4.65 (1H, m), 4.61 – 4.47 (4H, overlapped), 4.33 (1H, dd, *J* = 11.0, 3.1 Hz), 4.06 (1H, dd, *J* = 9.6, 7.5 Hz), 3.90 (1H, t, *J* = 6.2 Hz), 3.19 – 3.02 (3H, overlapped), 2.77 (1H, dd, *J* = 13.7, 10.0 Hz), 1.97 (1H, m), 1.75 (1H, m), 0.90 (3H, d, *J* = 6.8 Hz), 0.64 (3H, d, *J* = 6.7 Hz), 0.61 (3H, d, *J* = 6.8 Hz), 0.57 (3H, d, *J* = 6.7 Hz), 0.51 (3H, d, *J* = 6.8 Hz); ^13^C NMR (125 MHz, DMSO-*d*_6_) *δ* 173.0, 171.6, 171.4, 171.3, 171.1, 169.5, 168.5, 165.8, 165.5, 153.5, 153.4, 150.0, 149.9, 138.1, 137.9, 136.5, 132.3, 131.9, 129.8, 129.7, 129.6, 129.2, 129.1, 128.6, 127.7, 127.6, 127.6, 127.5, 126.8, 123.9, 122.9, 122.8, 121.4, 118.7, 111.8, 109.7, 65.1, 63.5, 60.0, 59.6, 55.7, 55.4, 52.9, 52.0, 48.5, 37.0, 30.5, 29.9, 26.9, 19.2, 19.2, 18.7, 18.1, 18.0; HRESIMS *m*/*z* 1087.4647 [M + H]^+^ (calcd for C_59_H_63_O_11_N_10_, 397.1054); 29% yield.

**Compound 19**: White, amorphous powder; ^1^H NMR (400 MHz, DMSO-*d*_6_): *δ* ppm 10.87 (1H, s), 8.54 – 8.43 (2H, overlapped), 8.33 – 8.21 (2H, overlapped), 8.11 (1H, d, *J* = 8.7 Hz), 8.06 (1H, d, *J* = 5.1 Hz), 7.89 (1H, d, *J* = 15.9 Hz), 7.74 (1H, d, *J* = 5.0 Hz), 7.56 (1H, d, *J* = 7.9 Hz), 7.50 (1H, d, *J* = 3.1 Hz), 7.32 (1H, d, *J* = 8.2 Hz), 7.26 – 7.19 (5H, overlapped), 7.17 – 7.11 (2H, overlapped), 7.06 (1H, t, *J* = 7.5 Hz), 6.96 (1H, t, *J* = 7.4 Hz), 6.12 (1H, d, *J* = 15.9 Hz), 4.71 (1H, m), 4.58 (1H, m), 4.50 (3H, overlapped), 4.44 (1H, dd, *J* = 12.3, 6.5 Hz), 4.15 (1H, m), 4.02 (1H, t, *J* = 8.9 Hz), 3.74 (1H, dd, *J* = 7.5, 5.1 Hz), 3.67 (1H, m), 3.43 (1H, m), 3.19 (1H, dd, *J* = 13.8, 4.7 Hz), 3.10 (1H, dd, *J* = 14.8, 7.2 Hz), 2.99 (1H, dd, *J* = 15.0, 7.4 Hz), 2.73 (1H, dd, *J* = 13.8, 10.3 Hz), 1.97 (1H, m), 1.78 (1H, m), 1.04 (3H, d, *J* = 6.7 Hz), 0.72 – 0.66 (6H, overlapped), 0.65 (3H, d, *J* = 6.8 Hz), 0.48 (3H, d, *J* = 6.7 Hz); ^13^C NMR (125 MHz, DMSO-*d*_6_) *δ* 172.5, 171.8, 171.5, 171.2, 171.1, 169.7, 169.4, 166.1, 139.3, 138.5, 138.3, 136.5, 132.5, 130.5, 129.6, 129.1, 128.5, 127.7, 126.7, 124.1, 121.4, 118.8, 118.6, 116.0, 111.7, 109.8, 61.5, 60.5, 56.5, 55.6, 55.2, 52.2, 48.5, 36.9, 30.9, 29.4, 19.7, 19.2, 18.6, 18.4, 17.9; HRESIMS *m*/*z* 913.3910 [M + H]^+^ (calcd for C_46_H_57_O_10_N_8_S, 913.3913); 26% yield.

**Table S1.** Derivatives **10**−**19** of asperversiamide A (**1**).

| No. | R_1_ | R_2_ | No. | R_1_ | R_2_ |
| --- | --- | --- | --- | --- | --- |
| **10** |  | R_1_ | **15** |  | R_1_ |
| **11** |  | H | **16** |  | R_1_ |
| **12** |  | R_1_ | **17** |  | H |
| **13** |  | R_1_ | **18** |  | R_1_ |
| **14** |  | H | **19** |  | H |

**Table S2**. Antitubercular activity of compounds **1**, **8**−**19** and previously reported **2**, **20**−**23** against *Mycobacterium tuberculosis* H37Ra

| No. | MICs (*μ*g/mL) | No. | MICs (*μ*g/mL) | No. | MICs (*μ*mol/L) |
| --- | --- | --- | --- | --- | --- |
| **1** | > 100 | **14** | 100 | **2** *^b^* | 100 *^b^* |
| **8** | > 100 | **15** | 100 | **20** *^c^* | 12.5 *^c^* |
| **9** | > 100 | **16** | 100 | **21** *^c^* | 12.5 *^c^* |
| **10** | 100 | **17** | 100 | **22** *^c^* | 12.5 *^c^* |
| **11** | 100 | **18** | 100 | **23** *^c^* | 12.5 *^c^* |
| **12** | 100 | **19** | 100 |  |  |
| **13** | 100 | rifampin*^a^* | 0.0156 |  |  |

*^a^* Rifampin was used as positive control; *^b^* Reported data (Hou et al. 2019); *^c^* Reported data (Chao et al. 2021).


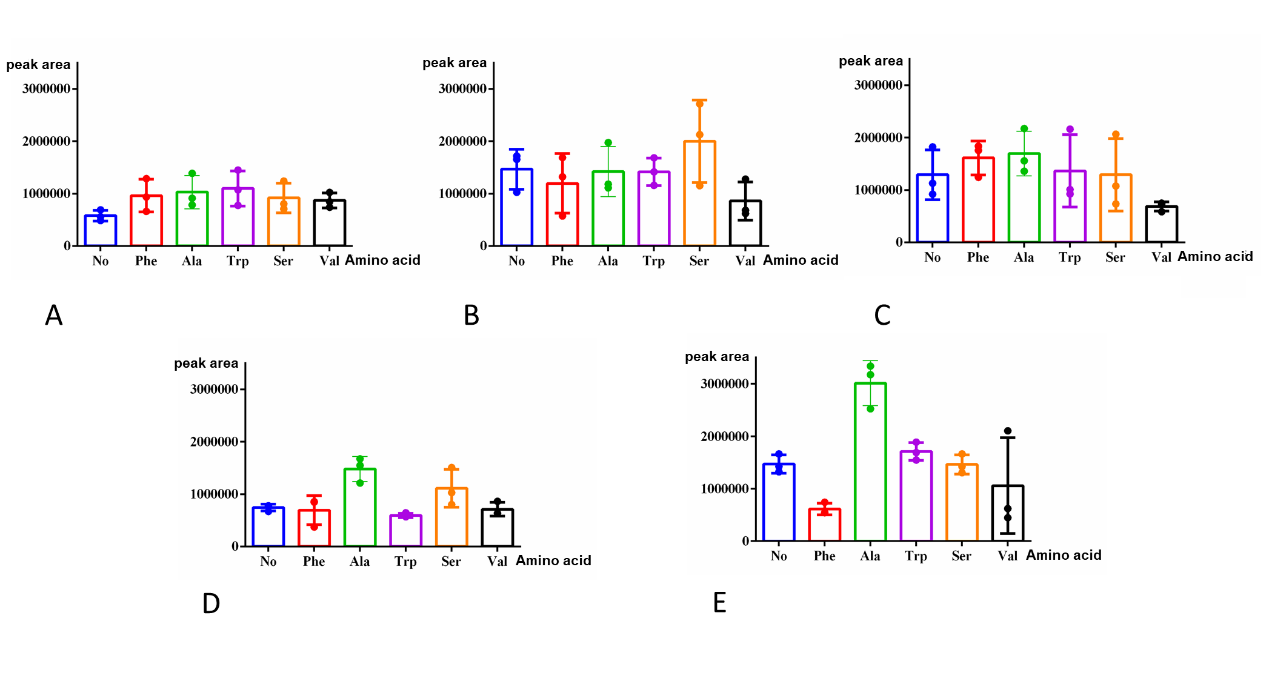


**Figure S1.** Production changes of asperversiamides A (**1**) after the addition of different amino acids at 4-8 weeks: (a) the fourth week; (b) the fifth week; (c) the sixth week; (d) the seventh week; (e) the eighth week.

**
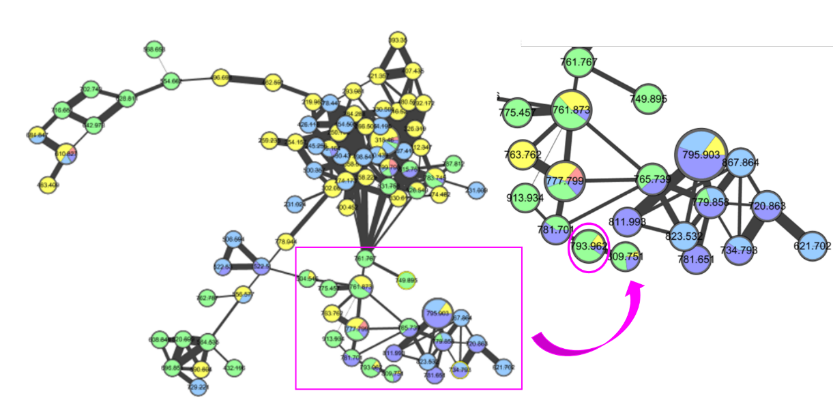
**

**Figure S2.** Clusters of cycloheptapeptide nodes from *Aspergillus* sp.


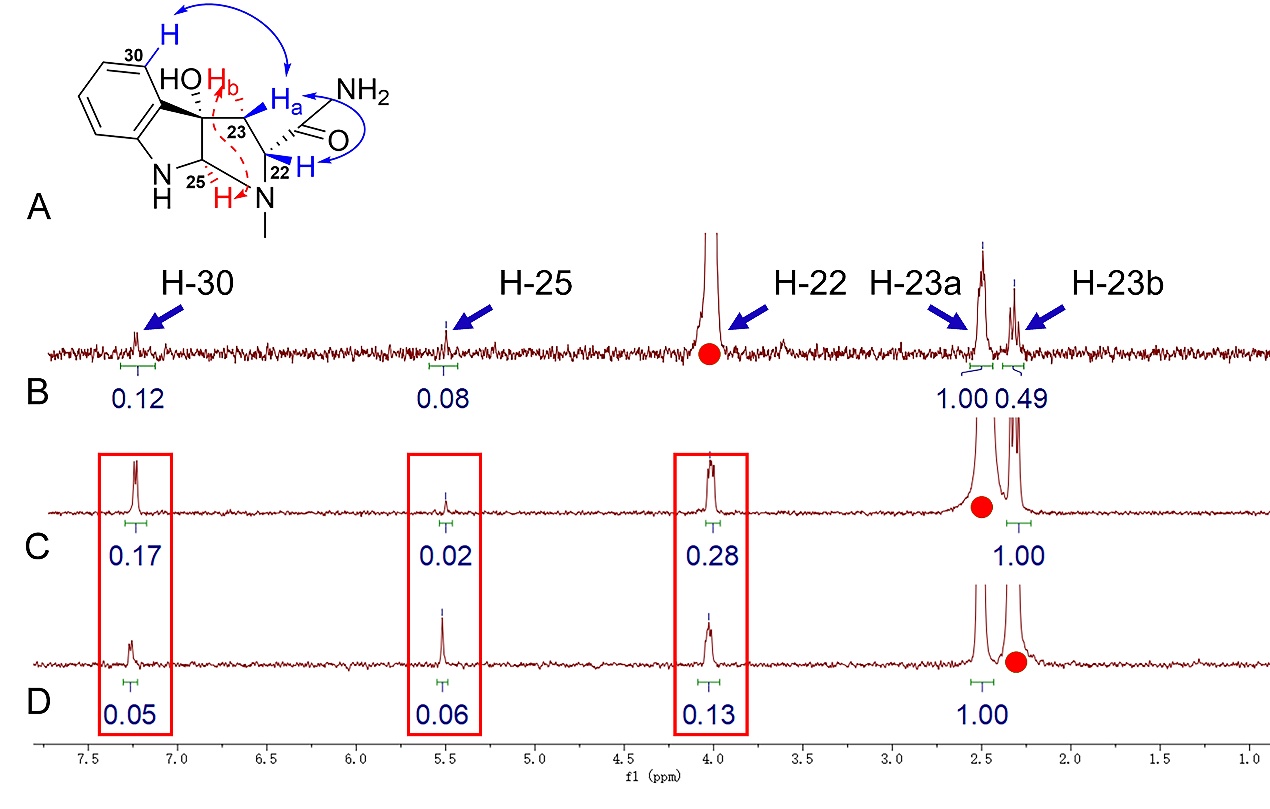


**Figure S3.** Selective NOE correlations observed for compound **8**: (a) pyrroloindoline fragment; (b) NOE spectrum, H-22 irradiated; (c) NOE spectrum, H-23a irradiated; (d) NOE spectrum, H-23b irradiated; Spectra were obtained in DMSO-*d*_6_ solution at 25 °C and 500 MHz.


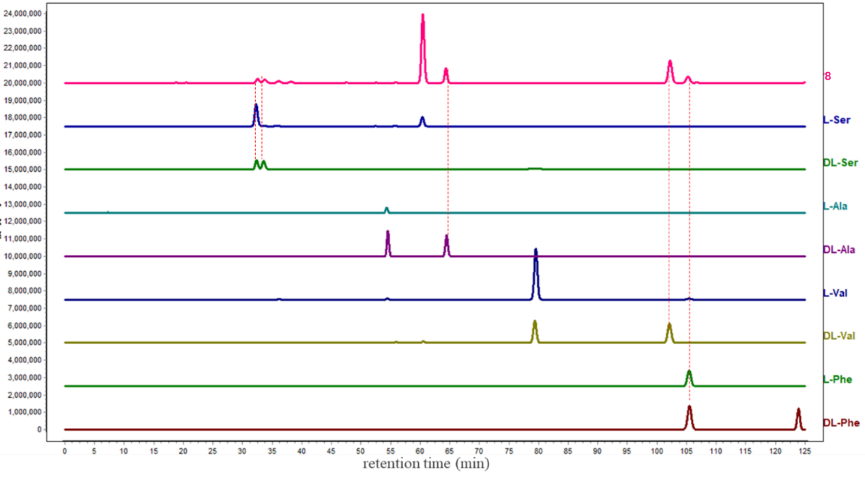


**Figure S4.** HPLC analyses of standard amino acids and hydrolysates of **8**.

| Cyclopeptides containing the pyrroloindoline motif | Source | Activity |
| --- | --- | --- |
| 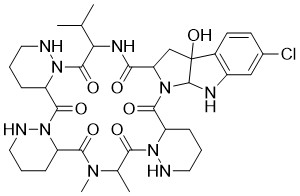  NW-G01 (Guo et al. 2009) | actinomycete *Streptomyces alboflavus* | antibacterial |
| 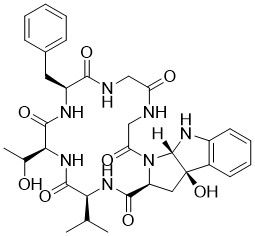  melicopteline C (Lee et al. 2020) | the leaves of *Melicope pteleifolia* | anti-influenza |
| 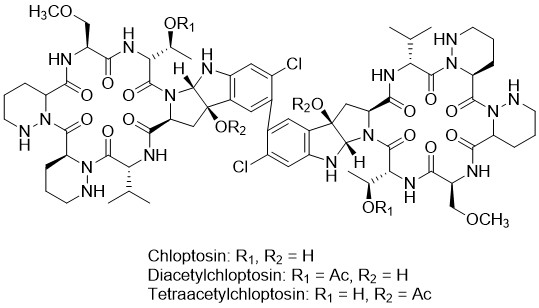  dimeric cyclohexapeptide chloptosin (Umezawa et al. 2000) | actinomycete *Streptomyces* MK498-98F14 strain | induce apoptotic and antimicrobial |
| 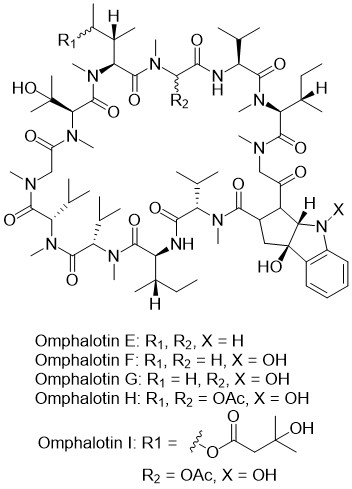  Omphalotins (Liermann et al. 2009) | basidiomycete *Omphalotus olearius* | Nematicidal |

**Figure S5.** Structure of cyclopeptides containing the pyrroloindoline motif from natural products.

**Figure S6.** HRESIMS of compound **8**.


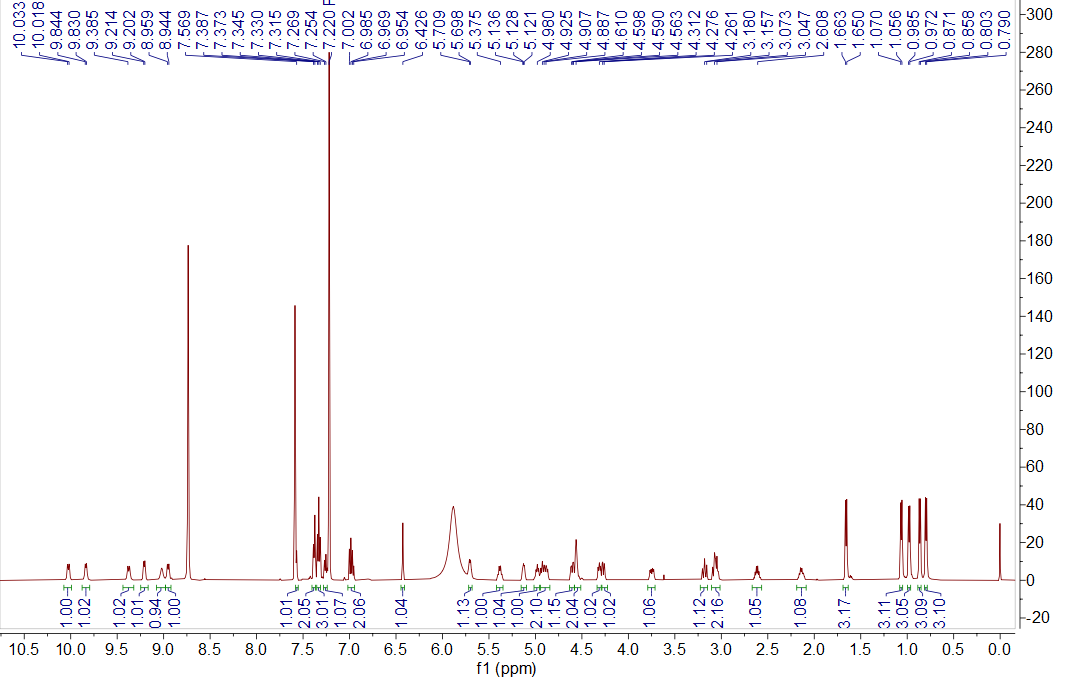
**Figure S7**. ^1^H NMR spectrum of compound **8** in pyridine-*d*_5_


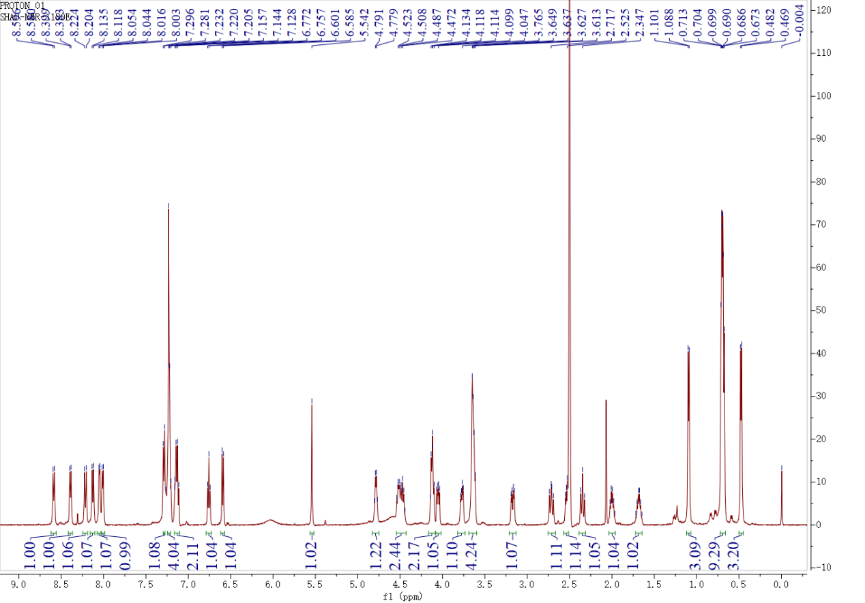


**Figure S8**. ^1^H NMR spectrum of compound **8** in DMSO-*d*_6_


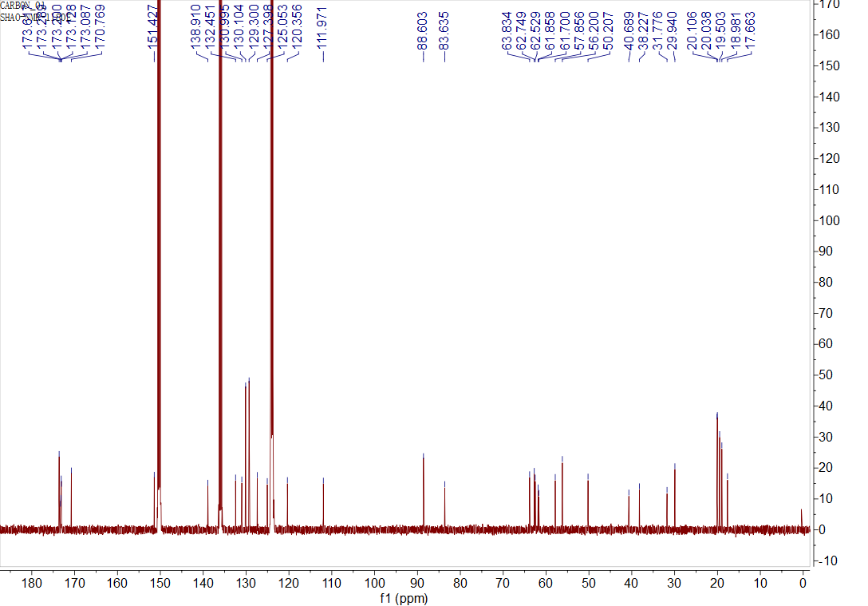


**Figure S9**. ^13^C NMR spectrum of compound **8** in pyridine-*d*_5_


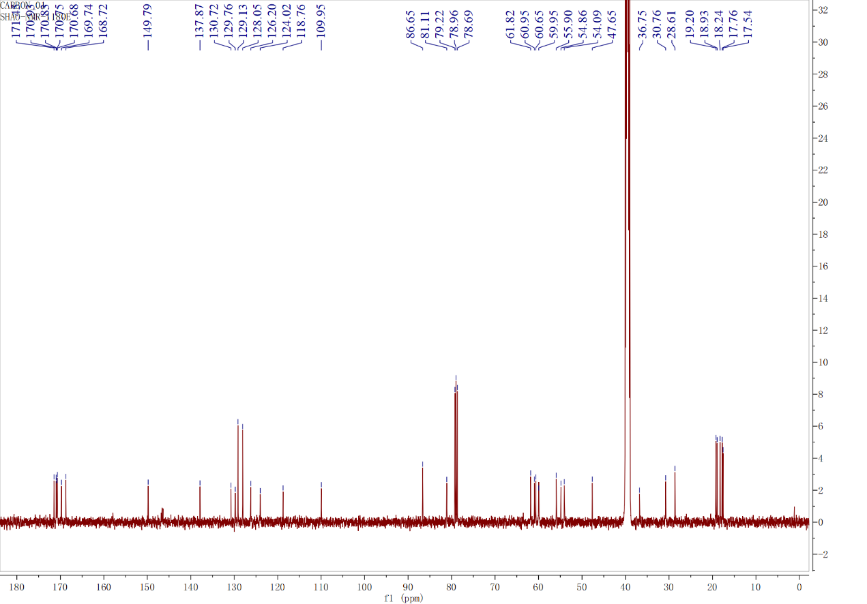


# **Figure S10**. ^13^C NMR spectrum of compound **8** in DMSO-*d*_6_


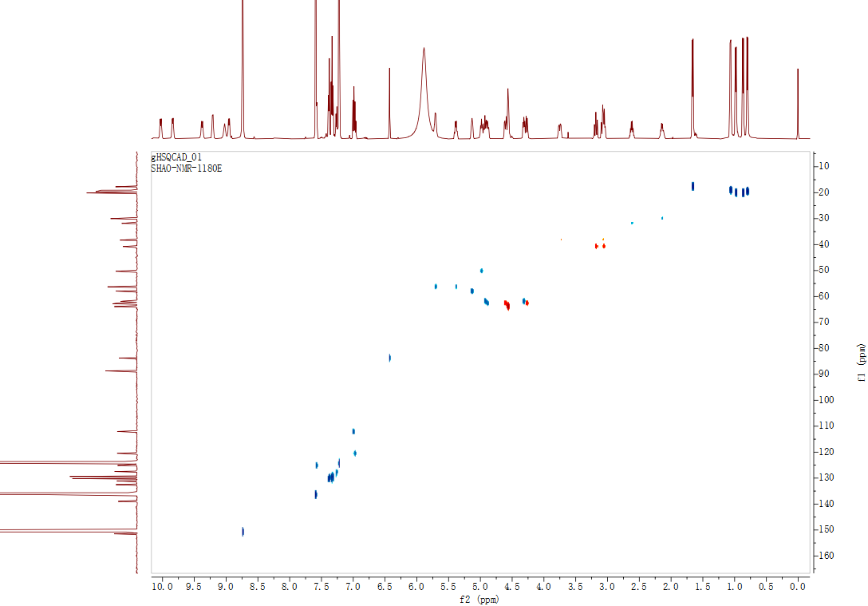


**Figure S11.** HSQC spectrum of compound **8** in pyridine-*d*_5_


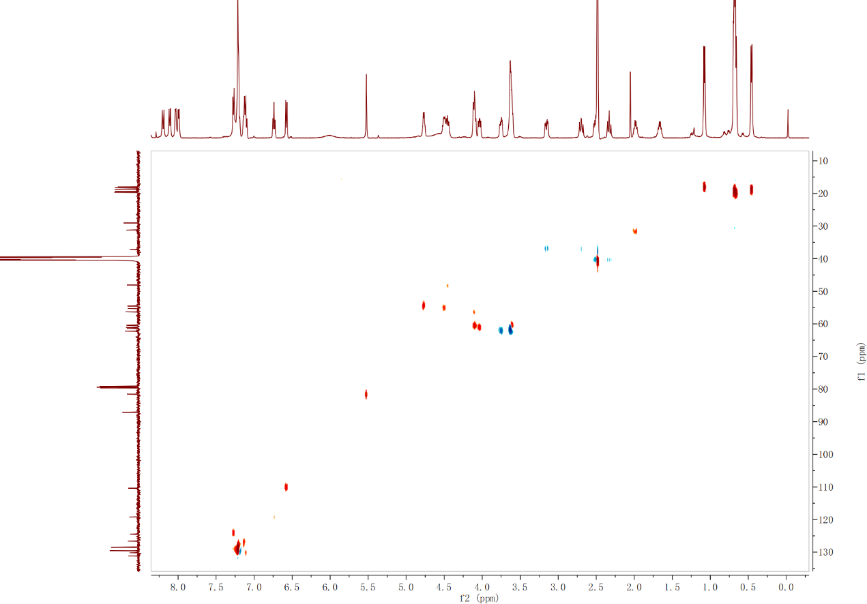


**Figure S12.** HSQC spectrum of compound **8** in DMSO-*d*_6_


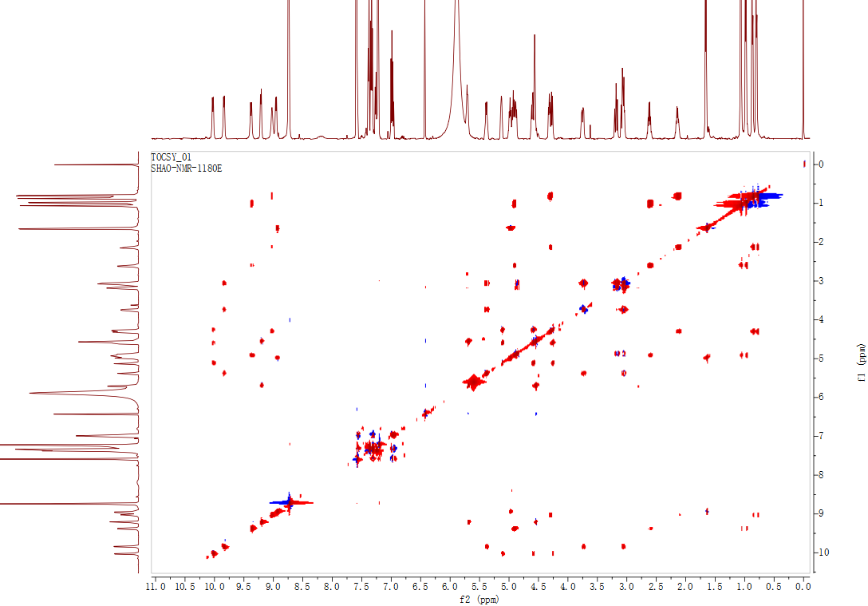


**Figure S13.** TOCSY spectrum of compound **8** in pyridine-*d*_5_


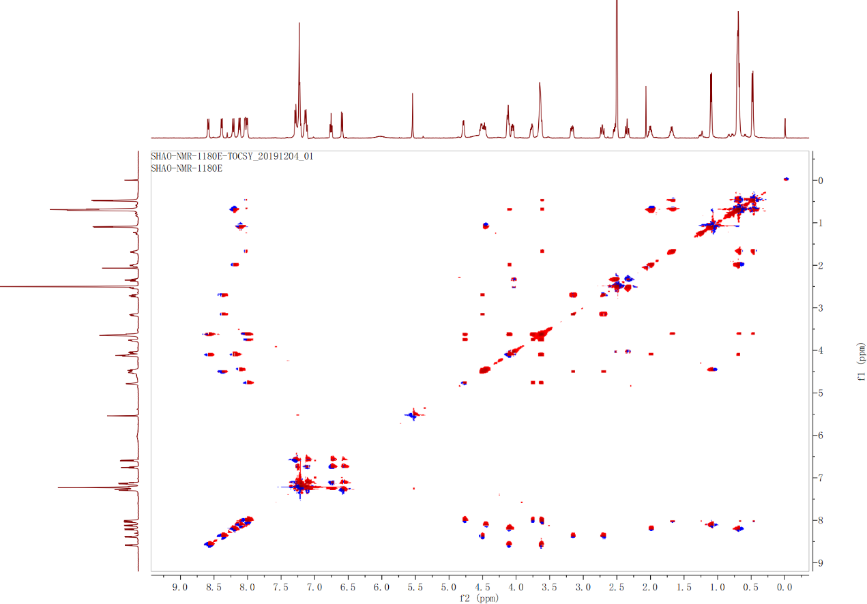


**Figure S14.** TOCSY spectrum of compound **8** in DMSO-*d*_6_


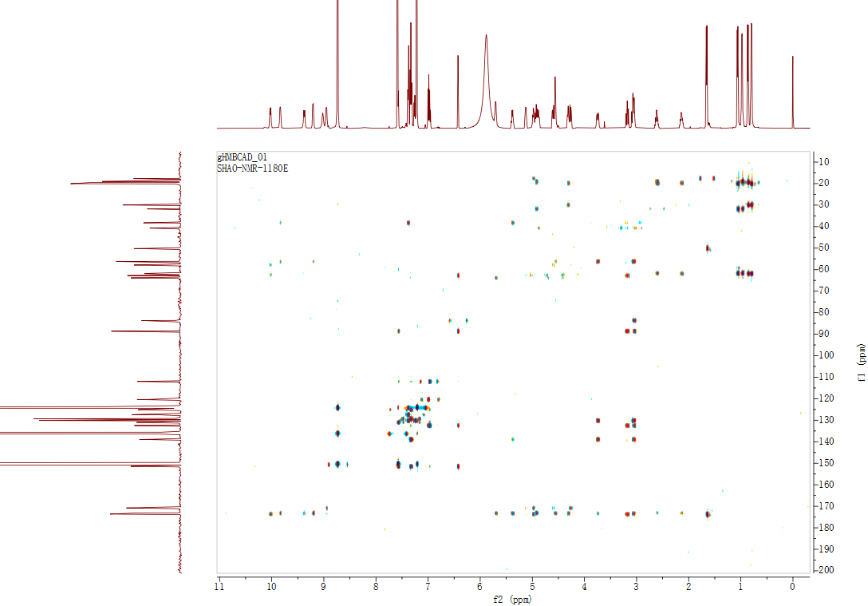


**Figure S15.** HMBC spectrum of compound **8** in pyridine-*d*_5_


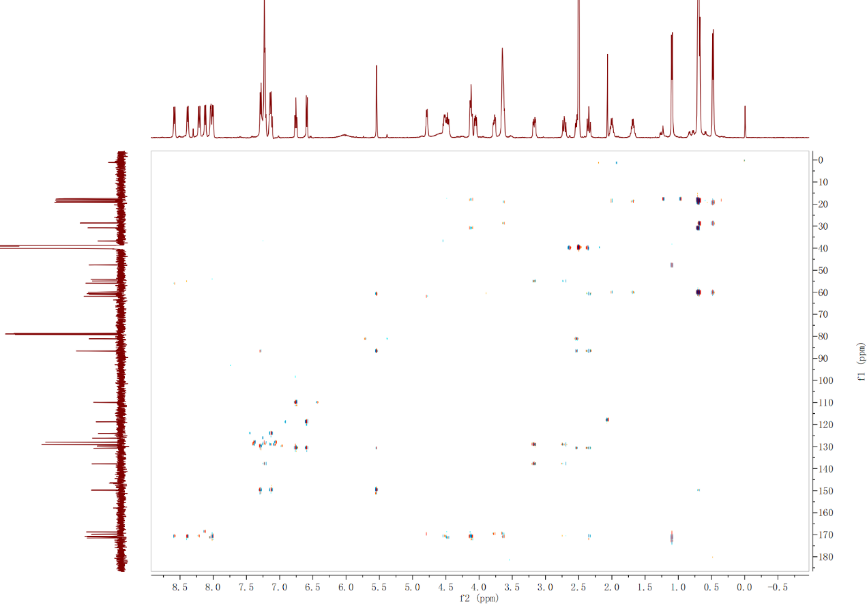


**Figure S16.** HMBC spectrum of compound **8** in DMSO-*d*_6_


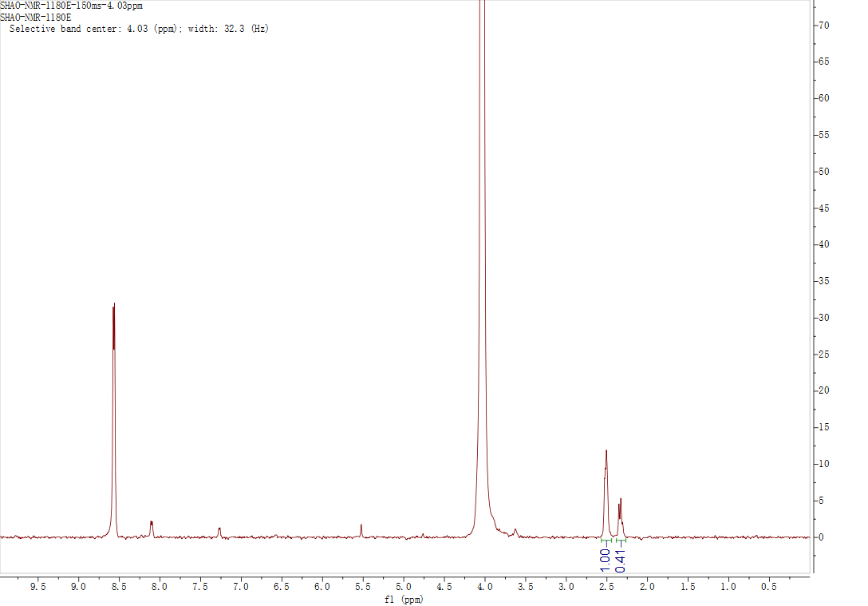


**Figure S17.** Selective NOE irradiation of H-22 with mixing time of 150 ms


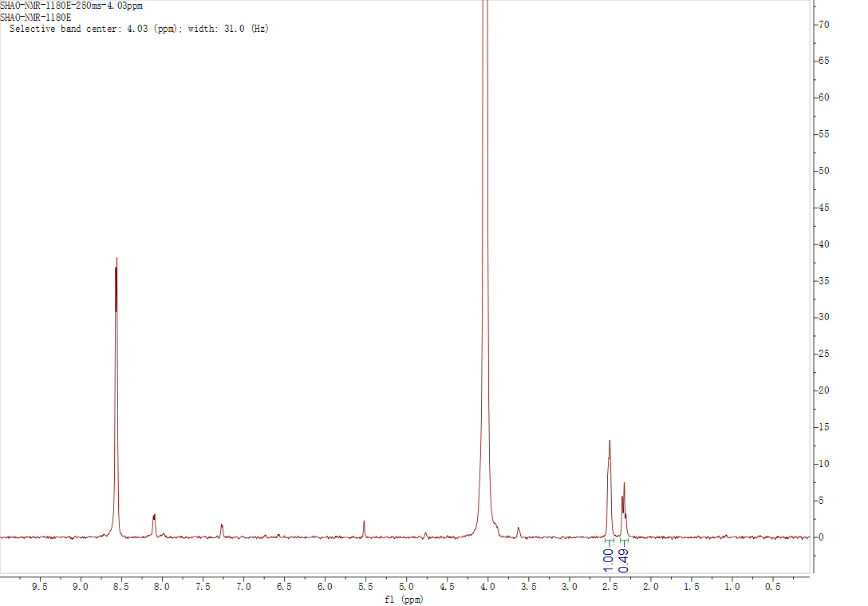


**Figure S18.** Selective NOE irradiation of H-22 with mixing time of 250 ms


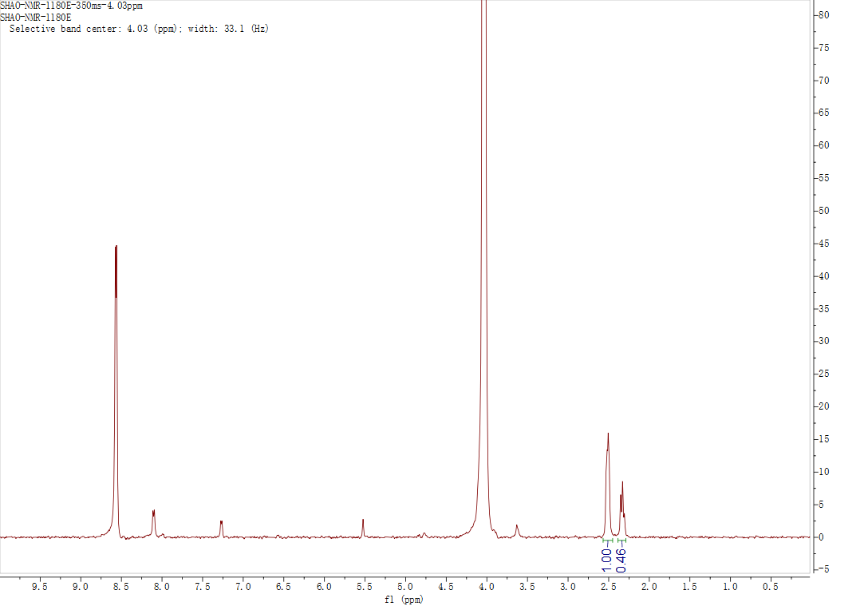


**Figure S19.** Selective NOE irradiation of H-22 with mixing time of 350 ms


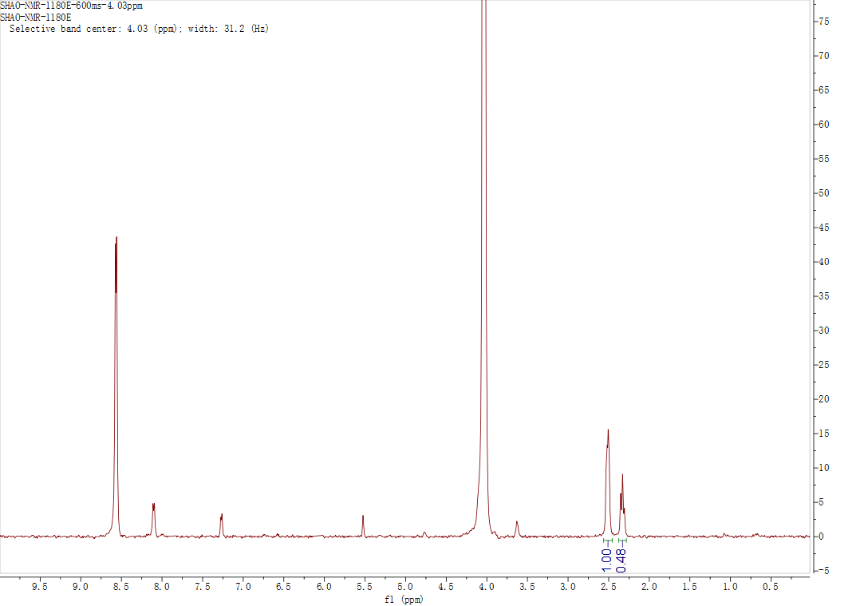


**Figure S20.** Selective NOE irradiation of H-22 with mixing time of 600 ms


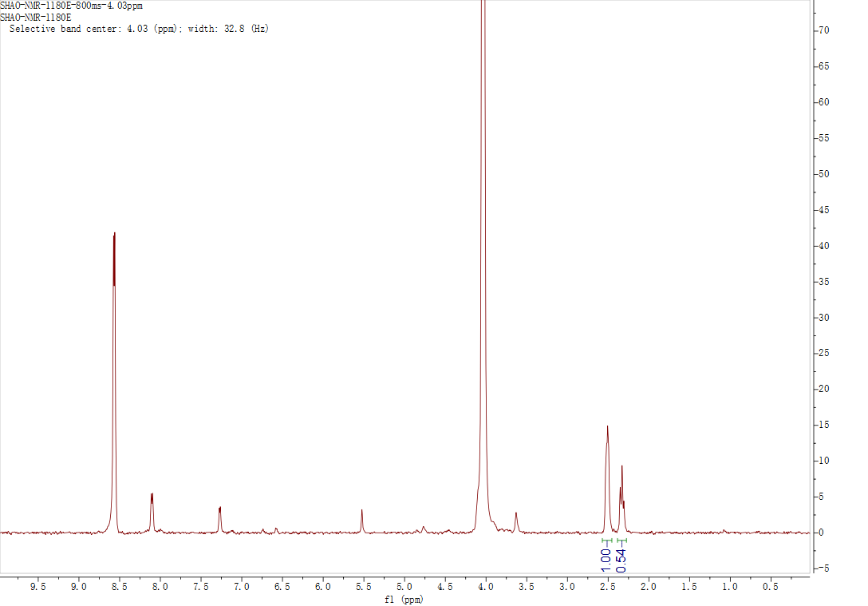


**Figure S21.** Selective NOE irradiation of H-22 with mixing time of 800 ms


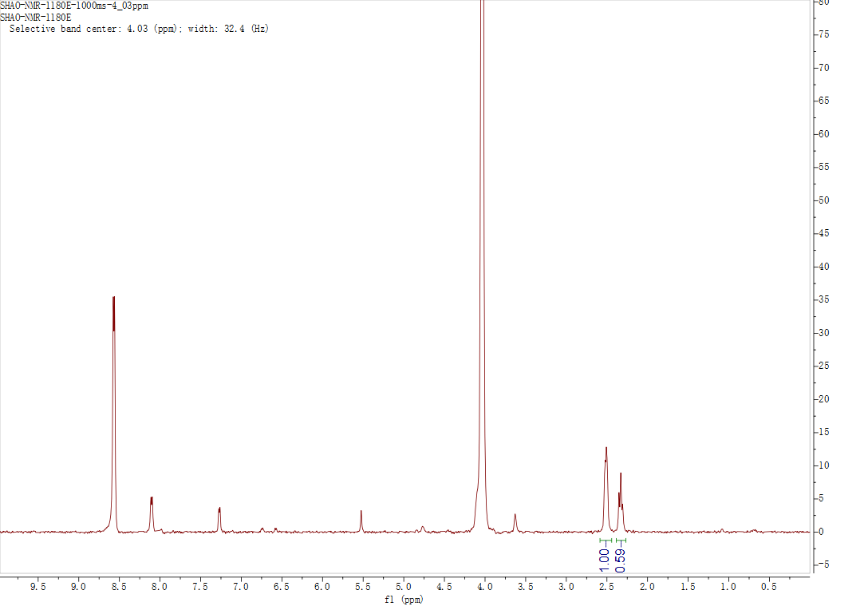


**Figure S22.** Selective NOE irradiation of H-22 with mixing time of 1000 ms

**Figure S23**. ESI-MS/MS of compound **8**.

**Figure S24**. ESI-MS/MS of compound **8**.

**Figure S25**. ESI-MS/MS of compound **8**.


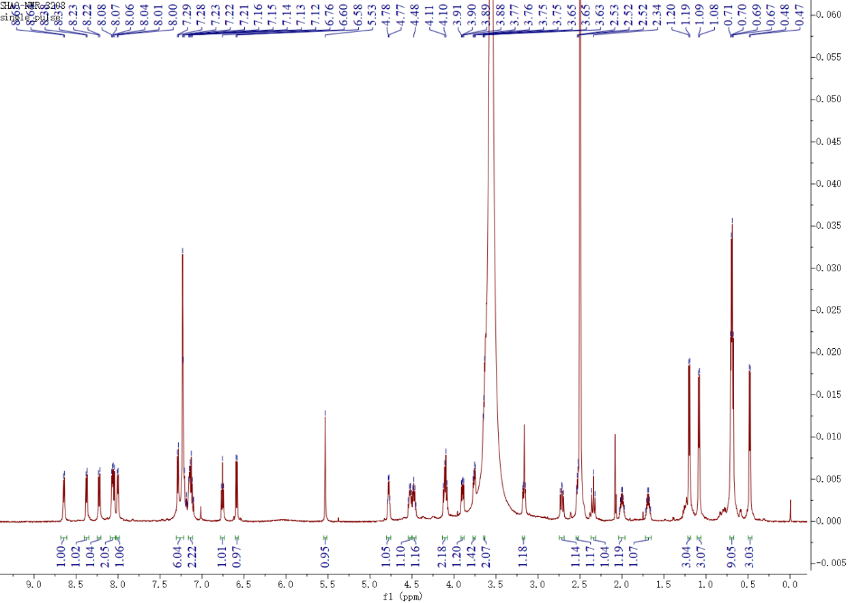


**Figure S26**. ^1^H NMR spectrum of compound **9** in DMSO-*d*_6_


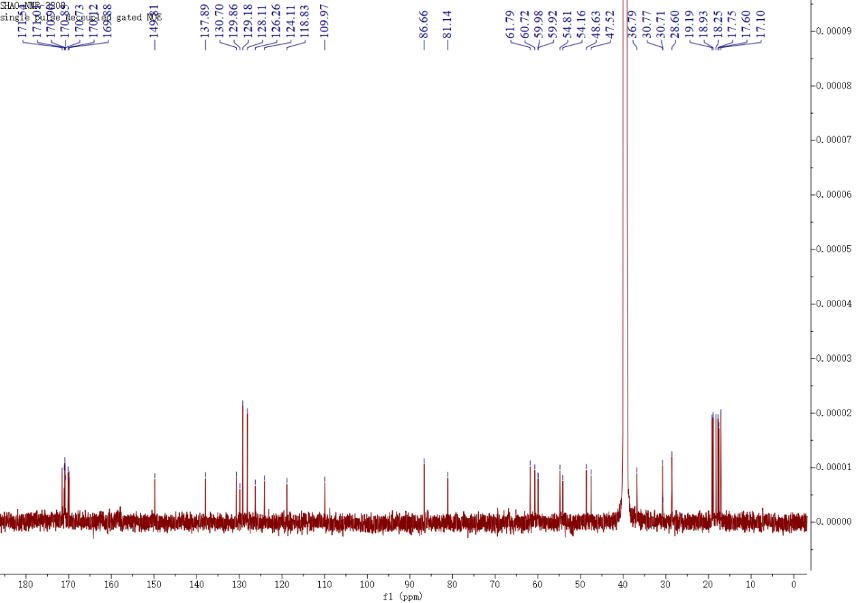


# **Figure S27**. ^13^C NMR spectrum of compound **9** in DMSO-*d*_6_

**Figure S28.** HRESIMS of compound **9**.


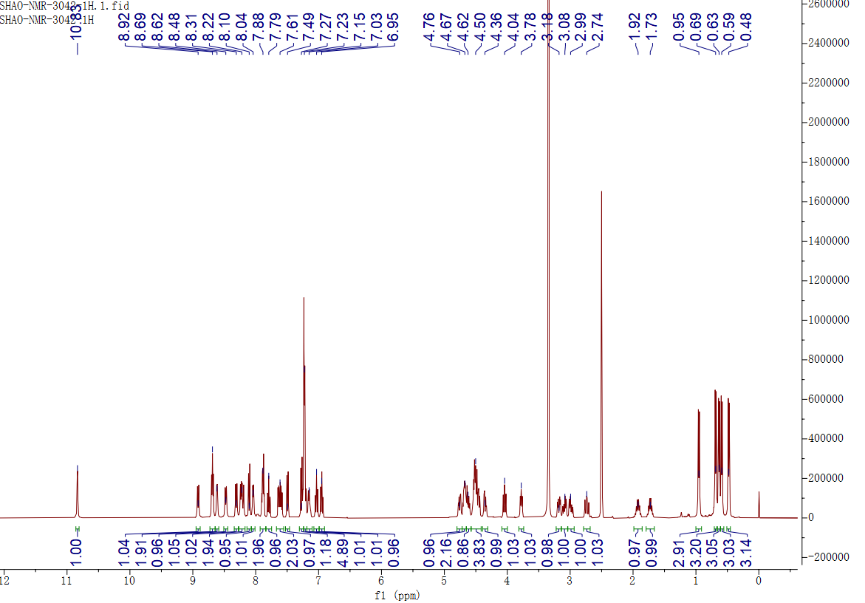


**Figure S29**. ^1^H NMR spectrum of compound **10** in DMSO-*d*_6_


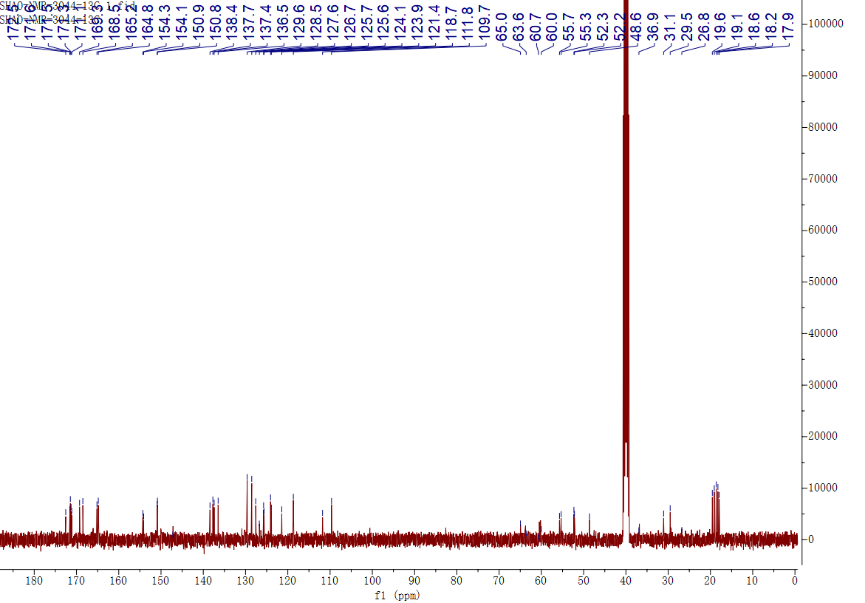


**Figure S30**. ^13^C NMR spectrum of compound **10** in DMSO-*d*_6_

**Figure S31.** HRESIMS of compound **10**.


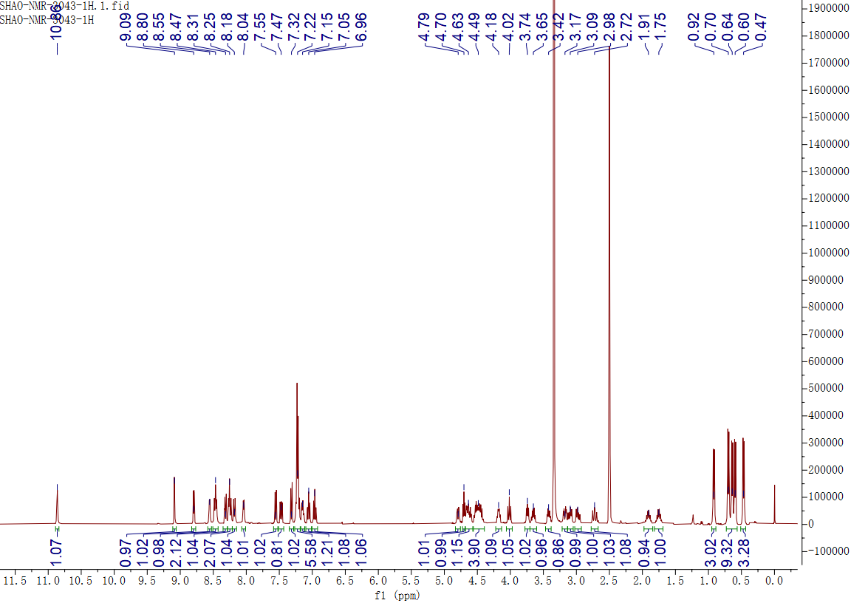


**Figure S32**. ^1^H NMR spectrum of compound **11** in DMSO-*d*_6_


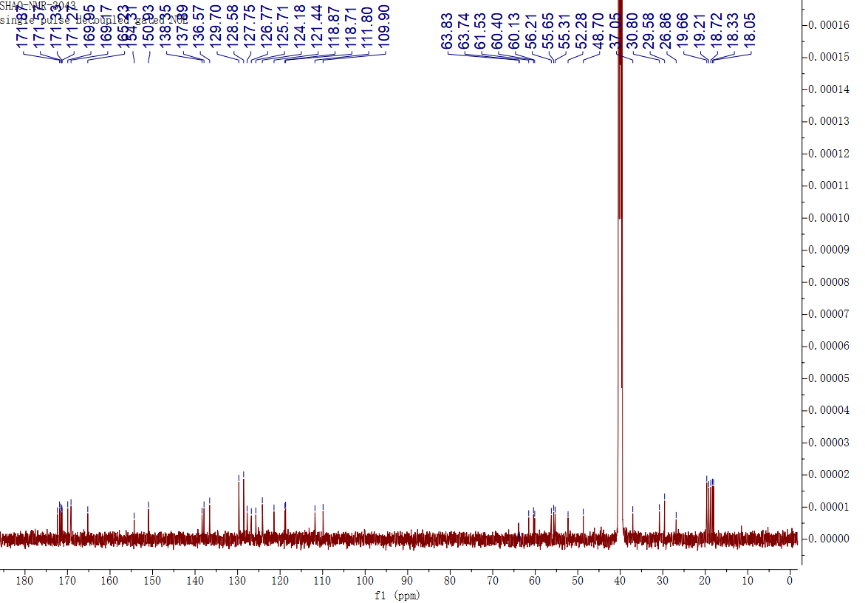


**Figure S33**. ^13^C NMR spectrum of compound **11** in DMSO-*d*_6_

**Figure S34.** HRESIMS of compound **11**.


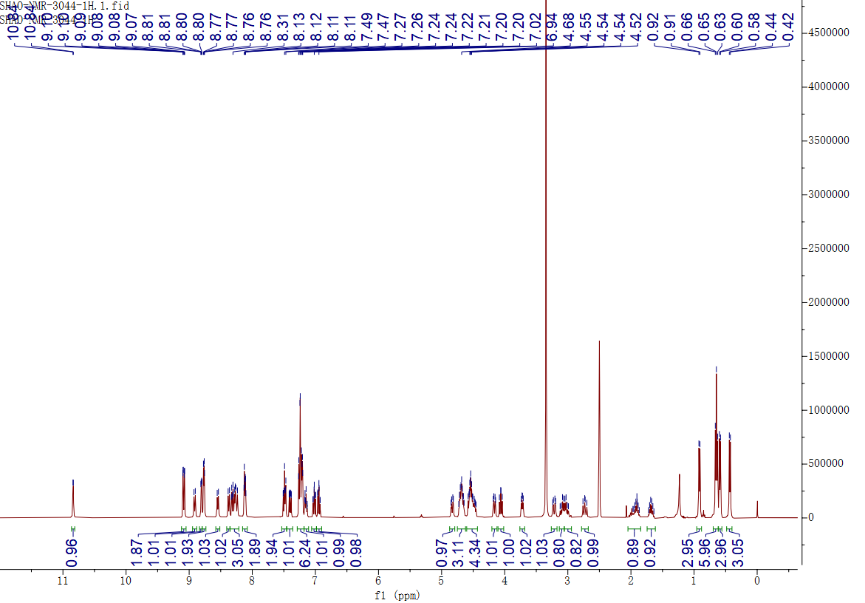


**Figure S35**. ^1^H NMR spectrum of compound **12** in DMSO-*d*_6_


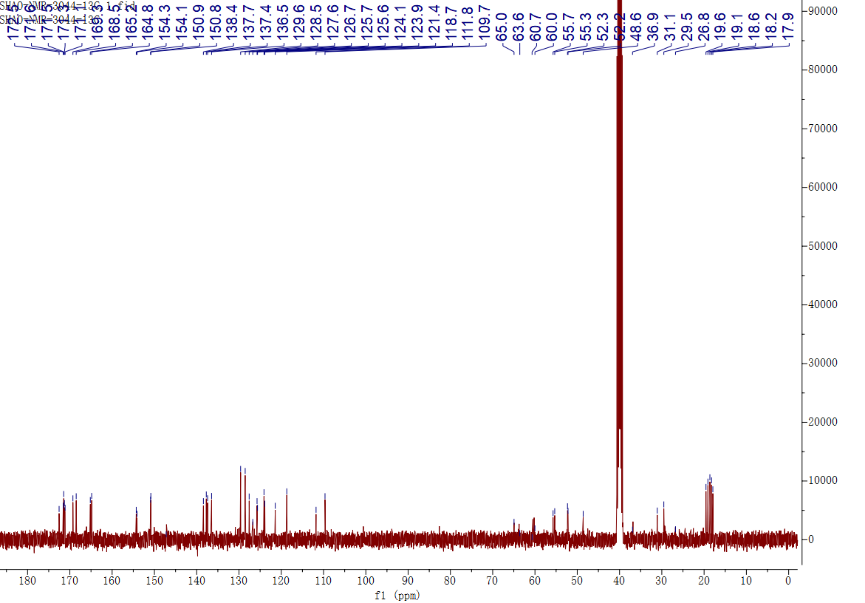


**Figure S36**. ^13^C NMR spectrum of compound **12** in DMSO-*d*_6_

**Figure S37.** HRESIMS of compound **12**.


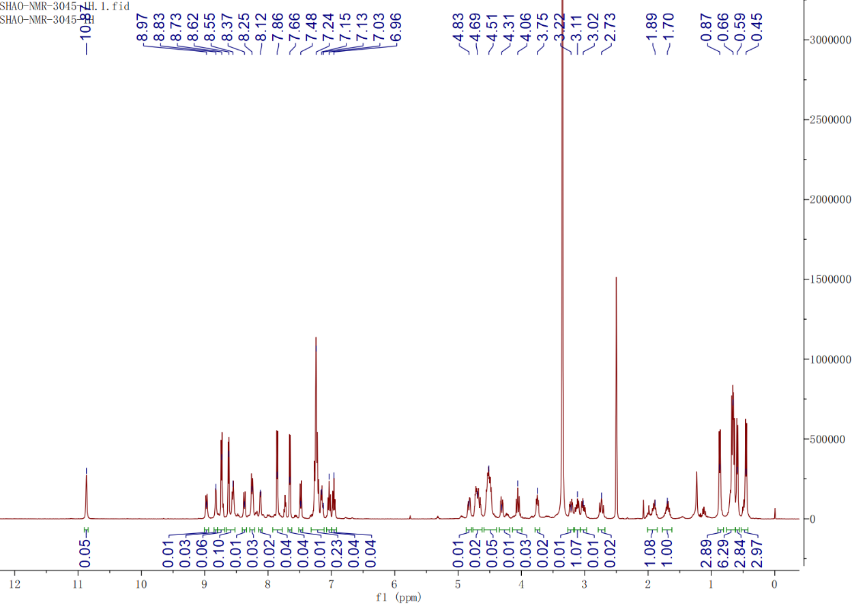


**Figure S38**. ^1^H NMR spectrum of compound **13** in DMSO-*d*_6_


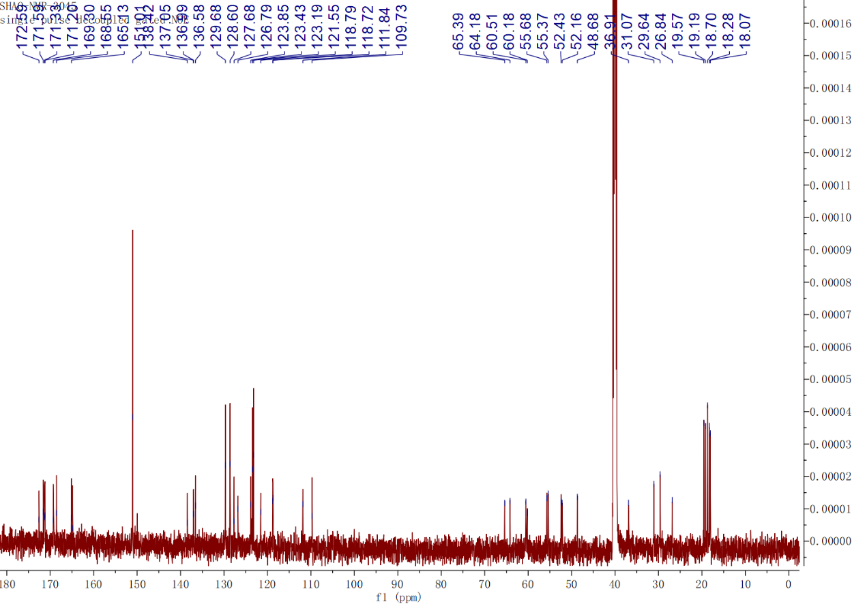


**Figure S39**. ^13^C NMR spectrum of compound **13** in DMSO-*d*_6_

**Figure S40.** HRESIMS of compound **13**.


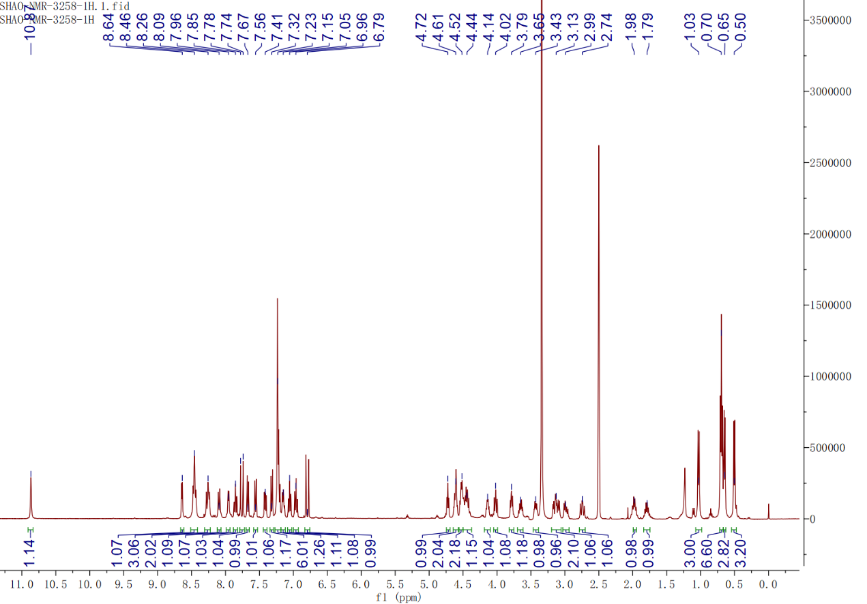


**Figure S41**. ^1^H NMR spectrum of compound **14** in DMSO-*d*_6_


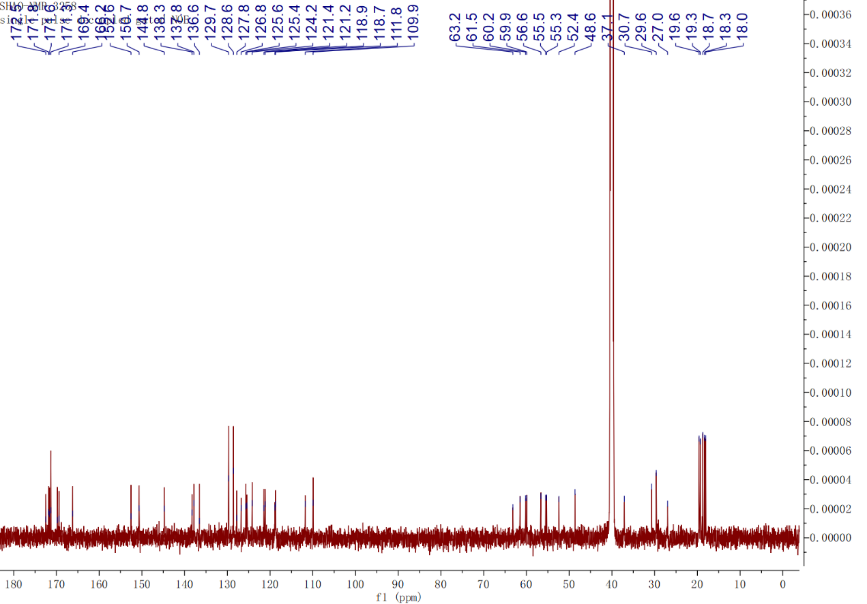


**Figure S42**. ^13^C NMR spectrum of compound **14** in DMSO-*d*_6_

**Figure S43.** HRESIMS of compound **14**.


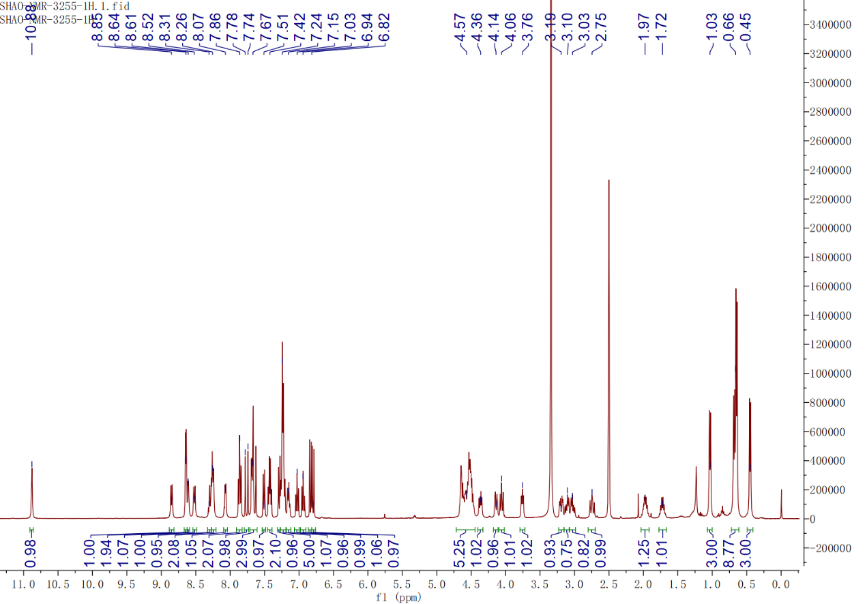


**Figure S44**. ^1^H NMR spectrum of compound **15** in DMSO-*d*_6_


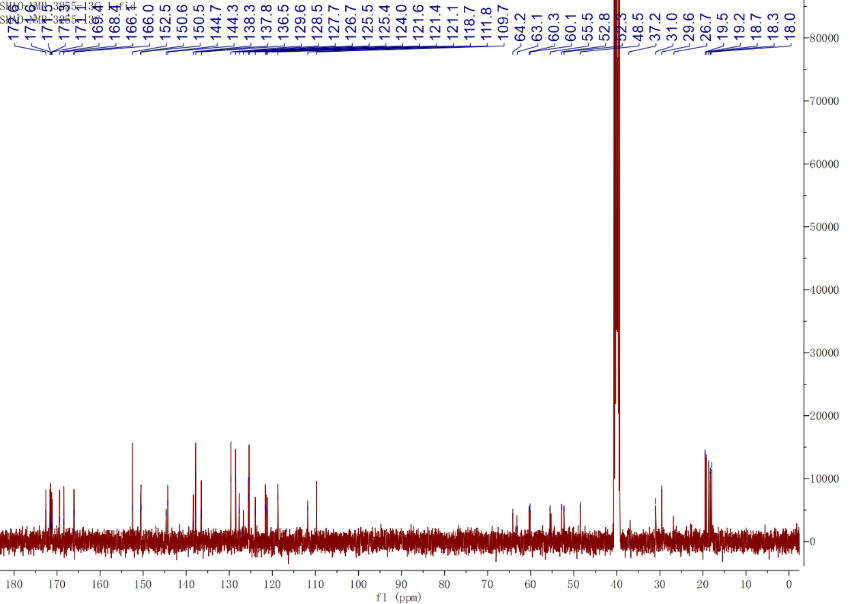


**Figure S45**. ^13^C NMR spectrum of compound **15** in DMSO-*d*_6_

**Figure S46.** HRESIMS of compound **15**.


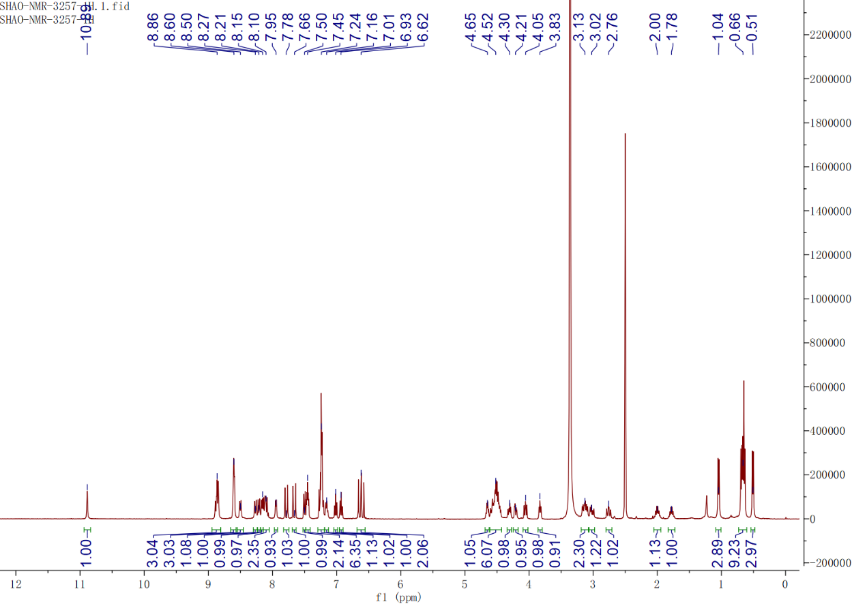


**Figure S47**. ^1^H NMR spectrum of compound **16** in DMSO-*d*_6_


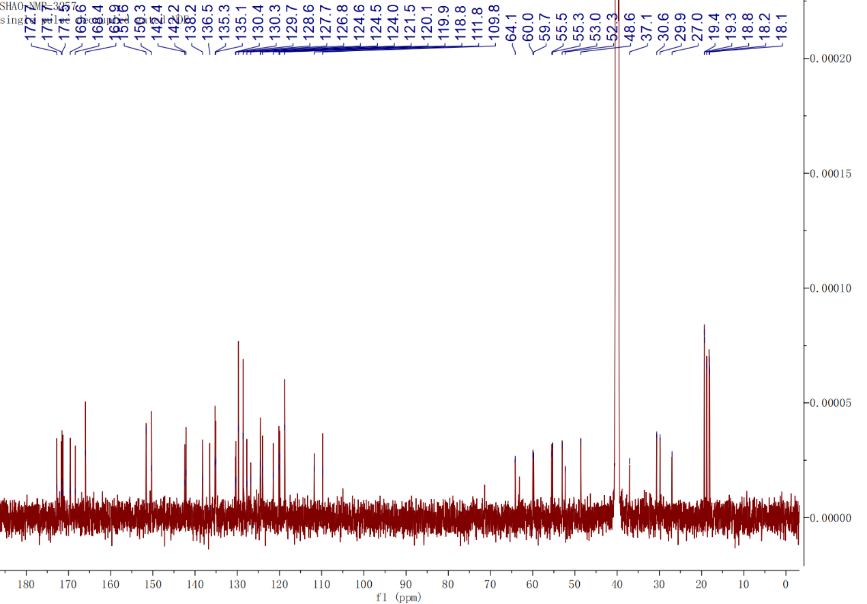


**Figure S48**. ^13^C NMR spectrum of compound **16** in DMSO-*d*_6_

**Figure S49.** HRESIMS of compound **16**.


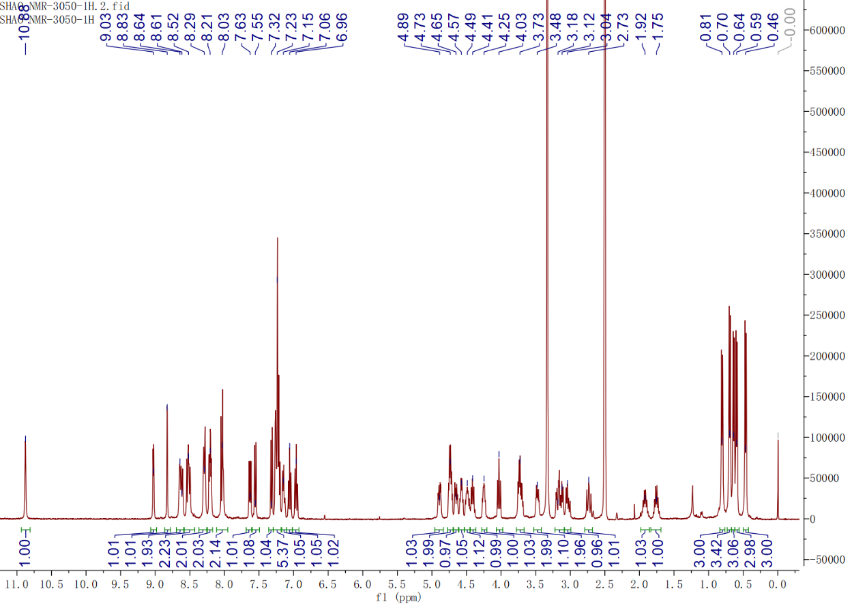


**Figure S50**. ^1^H NMR spectrum of compound **17** in DMSO-*d*_6_


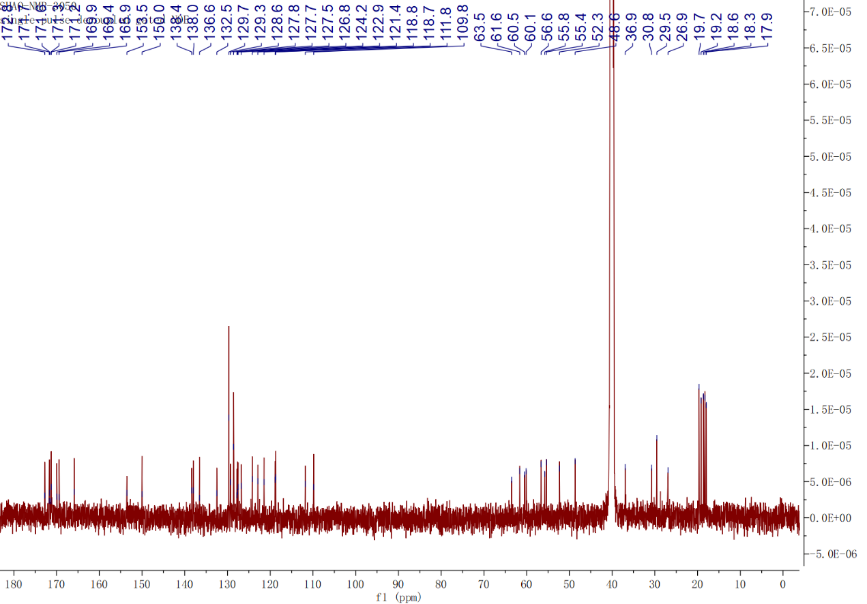


**Figure S51**. ^13^C NMR spectrum of compound **17** in DMSO-*d*_6_

**Figure S52.** HRESIMS of compound **17**.


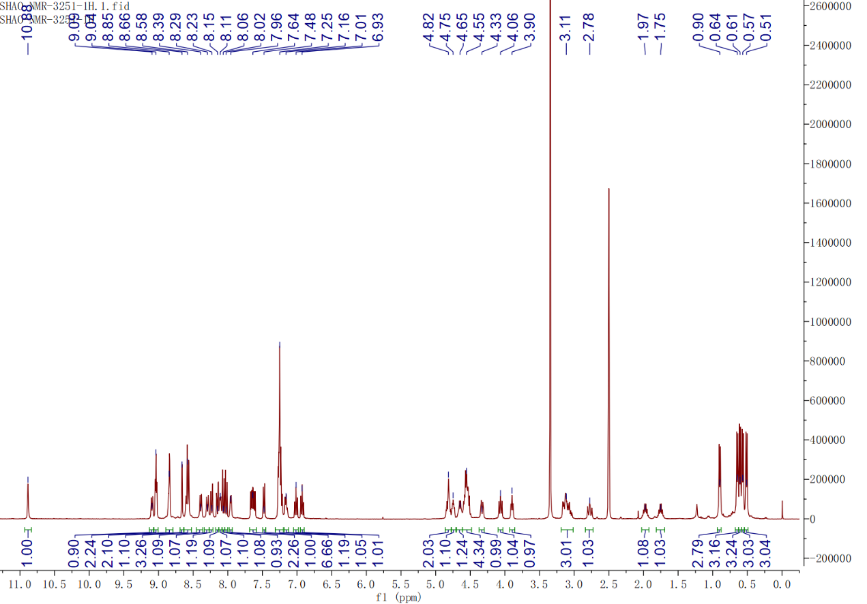


**Figure S53**. ^1^H NMR spectrum of compound **18** in DMSO-*d*_6_


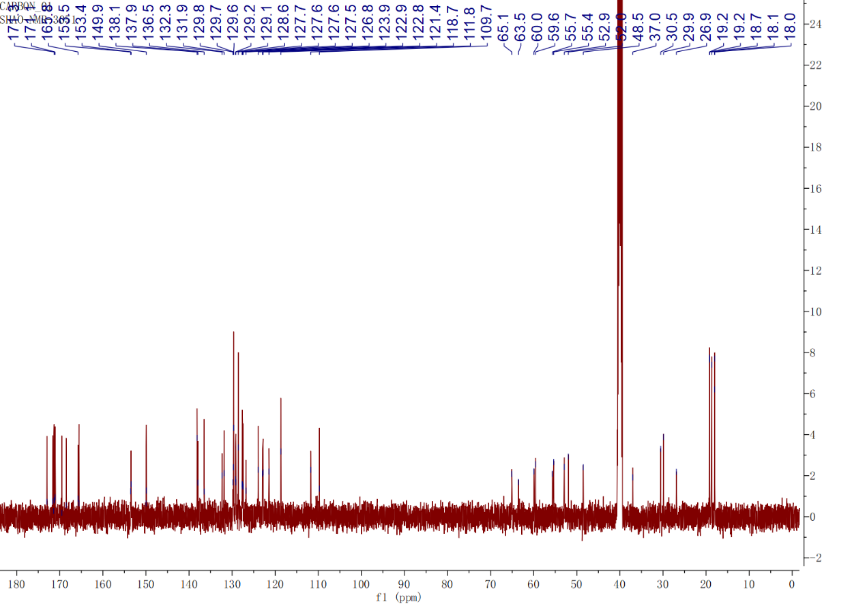


**Figure S54**. ^13^C NMR spectrum of compound **18** in DMSO-*d*_6_

**Figure S55.** HRESIMS of compound **18**.


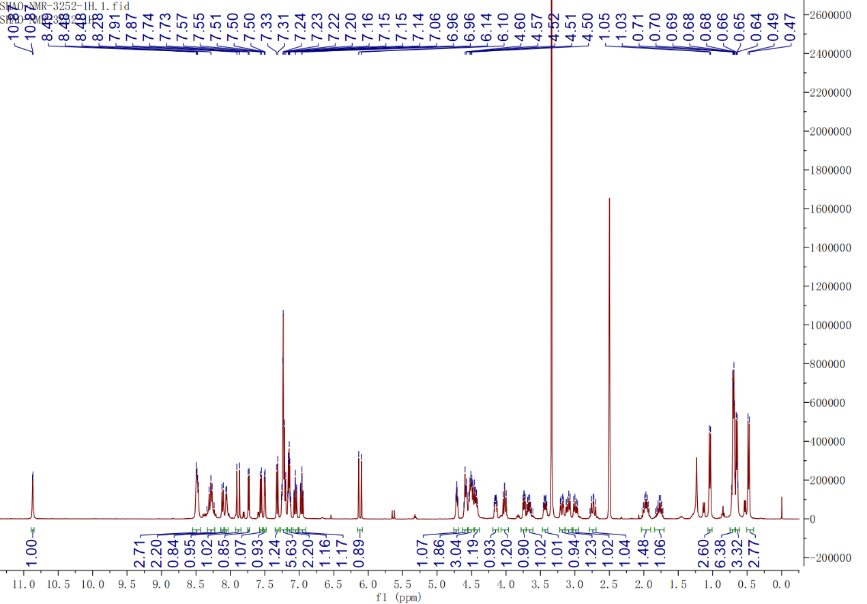


**Figure S56**. ^1^H NMR spectrum of compound **19** in DMSO-*d*_6_


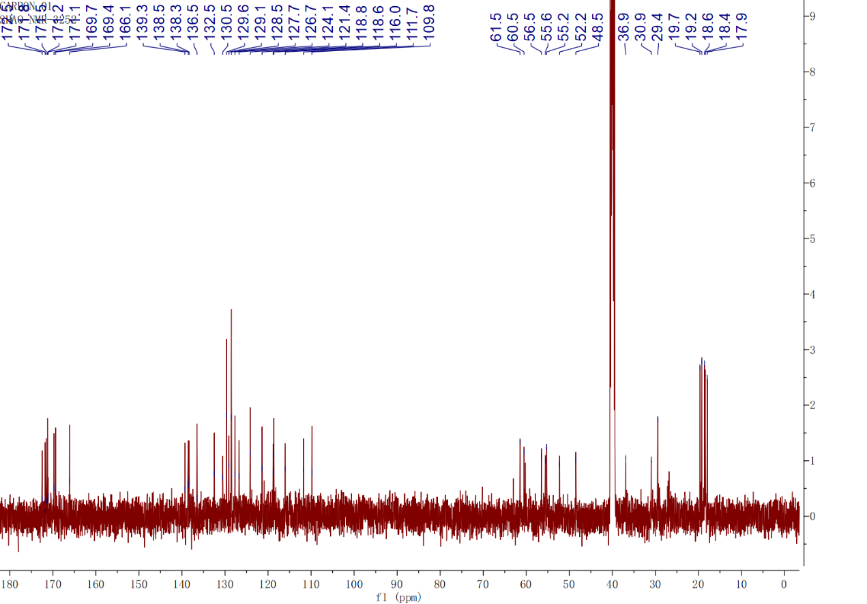


**Figure S57**. ^13^C NMR spectrum of compound **19** in DMSO-*d*_6_

**Figure S58.** HRESIMS of compound **19**.
